# Frequency of plastic occurrence varies across phylogeny but not ecology in seabirds

**DOI:** 10.1098/rsbl.2026.0079

**Published:** 2026-10-14

**Authors:** Stephanie Avery-Gomm, Nia Potapova, Chrissie Potter, Alexander L. Bond, Stephanie B. Borrelle, Jennifer L. Lavers, Steven J. Portugal, Hugh Possingham, Jennifer Provencher, Natalie Cooper

**Affiliations:** ^1^ School of Biological Sciences, University of Queensland, Brisbane, Queensland, Australia; ^2^ Wildlife and Landscape Science Directorate, Science and Technology Branch, Environment and Climate Change Canada, Ottawa, Ontario, Canada; ^3^ Science Group, Natural History Museum, London, UK; ^4^ Imperial College London, London, UK; ^5^ University College London, London, UK; ^6^ Bird Group, Natural History Museum, Tring, UK.; ^7^ BirdLife International, Pacific Secretariat, Suva, Fiji; ^8^ Adrift Lab, Underwood, Amarillo, USA; ^9^ Gulbali Institute, Charles Sturt University, Albury, Australia; ^10^ Department of Biological Sciences, University of Oxford, Oxford, UK; ^11^ University of Queensland, Brisbane, Australia

**Keywords:** diet, foraging strategy, body mass, pelagic specialists, marine debris, pollution, plastic ingestion, phylogenetic comparative methods

## Abstract

Seabirds are especially vulnerable to ingesting plastic pollution. However, we have a limited understanding of which traits might increase their risk of plastic ingestion. Here, we use a dataset of seabird plastic occurrence records (93 281 birds from 240 species) to investigate which species accumulate plastic and whether this is related to diet, foraging method, pelagic specialism, body mass or phylogenetic position. In our dataset, 182 of the 240 species (76%) contained plastic. Within orders, the highest levels were in pelicans (Pelecaniformes; 67%) and the lowest in penguins (Sphenisciformes; 5%). In our phylogenetically informed analysis, accounting for artefacts of study design (e.g. sample size, year, ocean, source, age class and sampling method), none of the ecological traits we tested had a consistent effect on plastic occurrence. Instead, phylogenetic relatedness was the best predictor in our models, reflecting the tight correlation between seabird phylogenetic groupings and their traits. This suggests that phylogenetic position may serve as a rapid way to identify species likely to accumulate plastic. Future research should focus on developing predictions of plastic accumulation risk for understudied species, implementing standardized methodologies and investigating species-specific morphological and environmental variables that may affect their risk of plastic accumulation.

## 1. Introduction

By 2030, plastic waste inputs to the oceans will increase by an order of magnitude compared with 2016 [1]. Ingestion of plastic by marine species is a serious consequence of plastic pollution [2] and can negatively impact ecosystem function [3–5]. The number of species affected by plastic pollution has doubled since the 1980s, with seabirds being the most well-documented animals known to ingest and accumulate plastics [6–8]. This is alarming because seabirds are among the most threatened groups of birds [9] and serve as key indicators of ecosystem health [10–12]. Plastic ingestion can result in physical and toxicological effects leading to reduced health, feeding capacity, growth and survival [4,13–15]. However, our understanding of how the acute and chronic impacts of plastic ingestion on individuals are connected to the effects of plastic at the population- or assemblage-level is limited [13,16,17]. It is, therefore, essential to understand which species are most at risk, to identify management priorities and develop mitigation strategies [18–21].

Plastic is already having detrimental effects on rare and threatened seabird species [22]. To determine which species are at most risk, we must improve our understanding of the factors that influence a species' vulnerability to ingesting and retaining plastic [23,24]. Numerous factors influence the likelihood that seabirds will ingest plastic. These include diet, use of anthropogenic food sources, foraging method (i.e. how they feed and at what depth), the senses seabirds use to locate prey (e.g. visual, olfactory), and whether plastics resemble prey in shape, colour and/or smell [25–31]. Seabirds may also ingest plastic indirectly by eating prey that previously consumed plastic [32–34]. How different species deal with indigestible items (e.g. regurgitation via pellets vs breakdown and excretion) will also influence the amount of plastic in seabirds [35]. The geographic and taxonomic constraints of past studies have, until recently, precluded a global synthesis to explore plastic occurrence across seabird species [24,26,32,36,37].

This study collates the largest dataset of seabird plastic occurrence data to date (93 281 birds) and represents a comprehensive advance in our understanding of the underlying determinants of plastic occurrence for seabirds. Using a phylogenetically informed approach, we (i) explore coverage of plastic occurrence research across seabird taxonomy and phylogeny, identifying groups that have been overlooked, and (ii) establish which traits contribute to the susceptibility of seabird species to ingesting and retaining plastic.

## 2. Material and methods

### (a) Data collection

We defined seabirds as species that depend on the marine environment for at least part of their life cycle (*n* = 387 species; see supplementary material for the full definition and species list) [38]. We followed the taxonomic treatment of [39] and used the phylogeny of [40] with minor modifications (supplementary material, table S1), pruned to seabirds. After excluding four species with uncertain phylogenetic placement, 383 species remained.

We collated the frequency of occurrence of plastic (FO; i.e. the proportion of birds in a study with evidence they had consumed plastic) in seabirds from the literature using Web of Science and Google Scholar (see supplementary material for details). The final dataset contains 262 sources published between 1958 and 2024. Although FO is a relatively crude measure, it was the most widely available across studies [6,41]. From each publication, we extracted the species, number of individuals sampled, number of individuals with plastic, age class of sampled birds (nest-bound, sub-adult/adult, adult, sub-adult or mixed), year(s) in which sampling was conducted, sampling method (bolus, brushing (i.e. removing prey items with a paintbrush; [42]), lavage, necropsy, regurgitation or droppings) and ocean basin (Arctic, Indian, North Atlantic, North Pacific, South Atlantic, South Pacific, or Southern Oceans). Several sources separated birds into smaller subgroups; however, in our analyses, we treated observations as replicates if they were collected for the same species, age class, publication, ocean basin, sampling method and year, using exact matching and aggregated the dataset accordingly, thereby avoiding duplicate observations.

Data on ecological traits were collected for each species from [43] and [44,]. We focused on traits we theorized could increase vulnerability to plastic ingestion. These were diet, use of anthropogenic subsidies, foraging method, whether a species is a pelagic specialist, and body mass. Traits were recorded at the species level and ignored variation among populations. Diet was categorized using the presence or absence of prey types in diet descriptions: ‘carrion and non-fish vertebrates’, ‘cephalopods’, ‘crustaceans’, ‘fish’ and/or ‘other invertebrates‥ Species were classified as using anthropogenic subsidies in the form of refuse (i.e. association with landfills etc.) and/or fisheries (i.e. attending trawlers, foraging on fisheries discards or offal, fish farms etc.). Foraging method was categorized on the basis of the presence or absence of foraging methods: ‘aerial pursuit’, ‘bottom-feeding’, ‘diving’ (moving through the water powered by feet or wings), ‘plunging’ (including pursuit plunging and surface plunging from any height, or any submersion of head/body while feeding on the water's surface), ‘skimming’ (including hydroplaning), ‘surface seizing’ (including dabbling, dipping and pattering), ‘terrestrial feeding’ (hunting live prey or scavenging on land) and/or ‘scavenging’ (on land or water). Species were classified as pelagic specialists if they predominantly forage in the pelagic zone according to [43].

Our final plastic occurrence dataset included 1763 valid records for 93 281 individual birds representing 240 of 387 seabird species (62%). In our models (see below), we simplified age class to nest-bound, free-flying (adult or sub-adult) and mixed (free-flying and nest-bound), then excluded records that only included nest-bound birds as these birds are not foraging for themselves, though we repeated the analyses, including these birds (supplementary material, table S9). We also excluded 10 species with incomplete ecological trait data. This left 1574 unique records of FO for 82 406 individual birds across 230 seabird species in our models (supplementary material, table S3). All data (including the raw data and cleaned data used in the analyses) are available from [45].

### (b) Statistical analysis

We used R version 4.5.3 [46] for all analyses, and the R packages: ape [47], coda [48], geiger [49], ggnewscale [50], ggtree [51,52], ggtreeExtra [52,53], here [54], Hmisc [55], janitor [56], MCMCglmm [57], patchwork [58], phytools [59], tidytree [52], tidyverse [60] and treeio [61]. All code is available on GitHub: https://github.com/nhcooper123/PhyloPlastics and archived at [62].

We first investigated the current state of plastic occurrence research in seabirds. We defined overlooked species as those with no records in our dataset, and well-studied species as those with at least 100 birds sampled across at least 3 records (i.e. three different studies, sampling locations, and/or years). For the species in our dataset, we calculated the mean and standard deviation of FO, weighted by the sample size. We visualized these results on the phylogeny.

To evaluate which biological and ecological traits influence the frequency of plastic occurrence in seabirds, we ran phylogenetic generalized linear mixed models (pGLMM) with binomial errors. Our response variable jointly modelled the number of individuals reported with plastic (*n plastic*) and the number without plastic (*n total − n plastic*) for a species in a study. We included diet and foraging method as binary variables for each diet or foraging method category, whether a species was a pelagic specialist or not, adult body mass (standardized by two standard deviations to make it comparable with binary variables [63] thus) and median year of study (scaled and centred) as fixed effects. We also included four study-level variables as random effects: age class of sampled birds (free-flying or mixed), sampling method, publication and ocean basin. The models also included two random effects for species: one unstructured random effect of species identity and a second using a correlation structure based on the seabird phylogeny pruned to the 230 species in the analysis. Before model fitting, we checked that no variables had correlations of greater than or equal to 0.80 to reduce issues with multicollinearity (supplementary material, table S2). We were unable to effectively control for differences in plastic exposure, because most seabirds forage across large areas so feeding areas are unlikely to be the same areas where birds were sampled.

We fitted models using the R package MCMCglmm [57]. Models were run for 1 × 10^5^ Markov chain Monte Carlo (MCMC) iterations, including a burn-in of 5000 iterations and a thinning interval of 100 iterations. We chose weakly informative priors (random effects: inverse Wishart with *V* = 1, *v* = 0.02; fixed effects: normal with *μ* = 0, *σ*^2^ = 1 × 10^10^), and assessed model fit using MCMC diagnostics in the R package coda, i.e., traceplots, posterior distribution checks and effective sample size estimation [48]. Effective sample sizes were above 600 for all parameters (supplementary material, table S5). The mean, median, mode and the 95% highest posterior density (HPD) of phylogenetic signal (*λ*) were estimated by dividing the phylogenetic variance–covariance matrix by the sum of the phylogenetic variance–covariance matrices [64]. We repeated the analyses with a variety of priors to ensure prior specification did not strongly influence model outputs.

A limitation of FO is that it assumes the occurrence of plastic is equivalent to plastic ingestion; in reality, FO reflects both ingestion and retention of plastic [35]. Species which regurgitate pellets [43] will have lower levels of plastic in their stomachs, but may still ingest as much plastic as species that retain plastic and slowly excrete it. No comprehensive data on gut retention rate are available for all species in our study, but this trait is phylogenetically conserved. Therefore, we repeated our analyses for Charadriiformes (*n* = 74; mostly pellet-producing) and Procellariiformes (*n* = 101; gut retention) separately to identify differences from the global model that may be related to how species deal with indigestible items. We also performed several additional sensitivity analyses (see supplementary material).

## 3. Results

Our plastic occurrence dataset contained 240 species (62% of seabird species; figure 1; supplementary material, table S3); 143 (38% of seabird species) species had no records in our dataset. Of the 240 species with plastic occurrence data, 182 (76%) contained plastic and 58 did not. Sample sizes for species ranged from 1 to 62 studies (median = 2; supplementary material, figure S1A), and 1 to 6673 birds (median = 77; supplementary material, figure S1B). Ninety-eight species were well-studied (i.e. at least 100 birds sampled across at least 3 records; supplementary material, figure S2) and 90 of these contained plastic.

**Figure 1.**
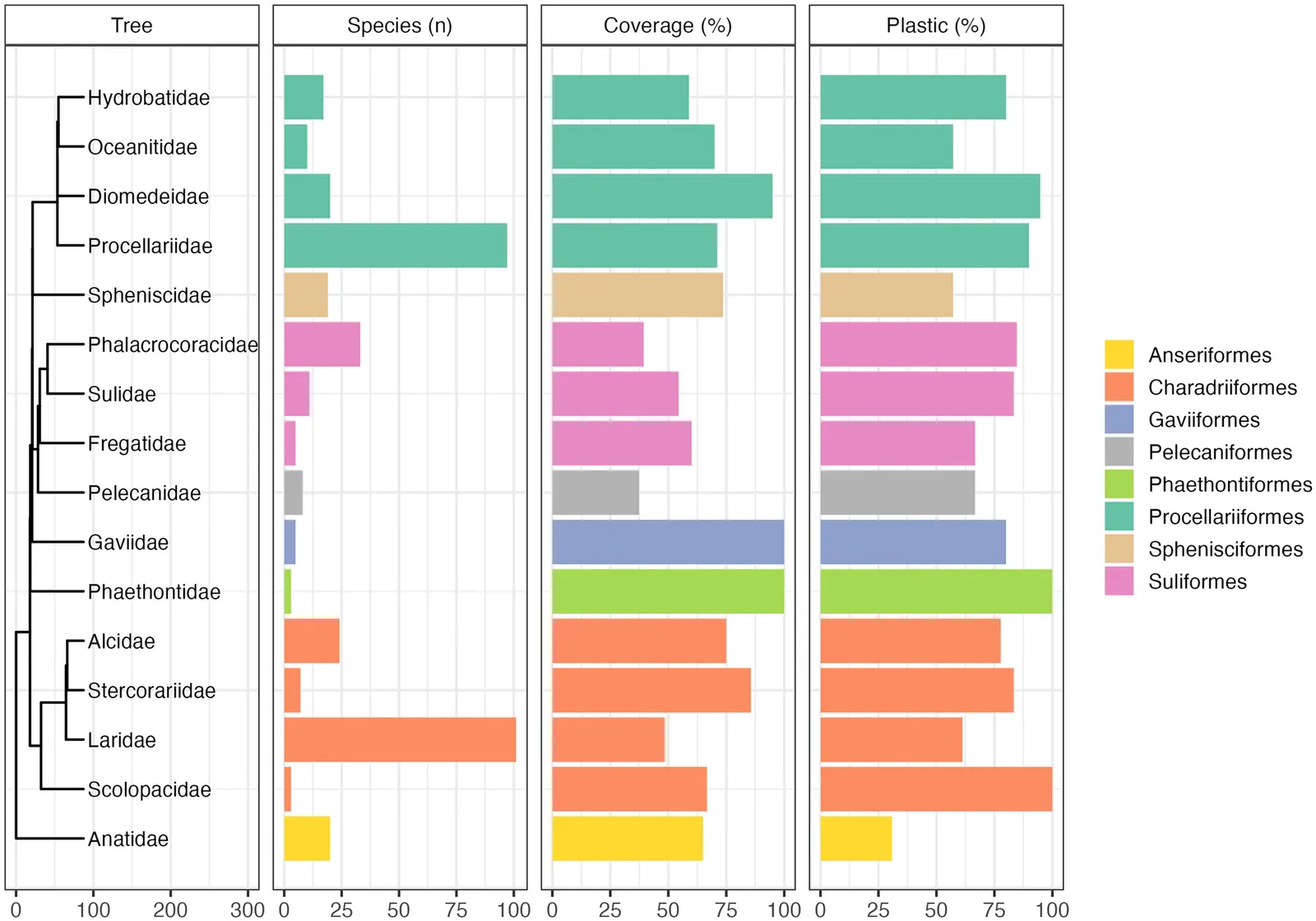
Family-level coverage of our plastic occurrence dataset. From left to right, the panels show the family-level phylogeny; the number of species in our dataset; the percentage species coverage; and the percentage of species containing plastic. Colours indicate the order.

Dataset coverage and FO varied across orders, families and ecological categories (figures 1 and 2; table 1; supplementary material, tables S3–S4, figures S2–S7). Species coverage across orders ranged from 37.5% in pelicans (Pelecaniformes) to 100% in tropicbirds (Phaethontiformes; though this order only contains three species), and within families from 39.4% in cormorants and shags (Phalacrocoracidae) to 100% in tropicbirds (Phaethontidae) and loons and divers (Gaviidae; supplementary material, table S3; figure 1). Pelicans (Pelecaniformes) had the highest weighted mean FO (66.7%; s.d. = 39.7%; *n*_species_ = 3; *n*_studies_ = 4; *n*_birds_ = 45), and penguins (Sphenisciformes) the lowest (5.15%; s.d. = 7.75%; *n*_species_ = 14; *n*_studies_ = 59, *n*_birds_ = 8326; figure 2; table 1). Four species have FO of 100% (*Leucophaeus atricilla* (laughing gull; *n*_studies_ = 1; *n*_birds_ = 13)*, Pelecanus occidentalis* (brown pelican; *n*_studies_ = 2; *n*_birds_ = 26)*, Pseudobulweria aterrima* (Mascarene petrel; *n*_studies_ = 1; *n*_birds_ = 1) and *Phalacrocorax sulcirostris* (little black cormorant; *n*_studies_ = 1; *n*_birds_ = 1)), and 37 species have FO greater than or equal to 50% (supplementary material, table S4).

**Figure 2.**
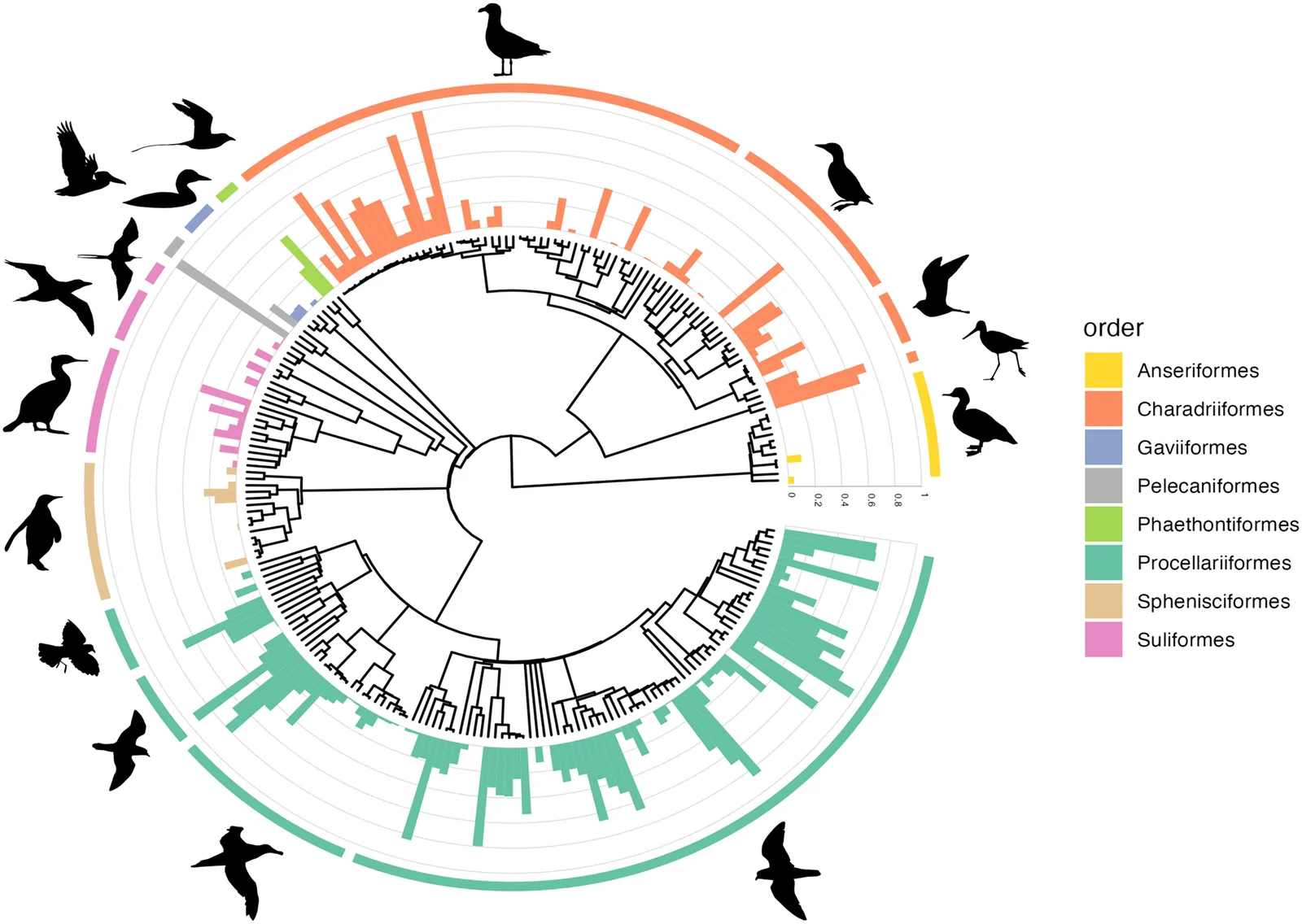
Weighted mean FO for the 230 species in our models. See table 1 for standard deviationss. Bars and clade labels are coloured by order. Families are indicated by silhouettes (from phylopic.org, CC0 1.0 license; see key in supplementary material). To aid readability, species names are omitted.

**Table 1.** Weighted mean and s.d. of FO across seabird orders and families.

**order**	**family**	**weighted mean FO (%)**	**weighted s.d. FO (%)**
Anseriformes	Anatidae	7.51	5.05
Charadriiformes	Alcidae	13.1	15.3
	Laridae	19.7	21.7
	Scolopacidae	39.6	0.14
	Stercorariidae	36.1	8.28
	ALL	21.9	19.3
Gaviiformes	Gaviidae	6.10	6.22
Pelecaniformes	Pelecanidae	66.7	39.7
Phaethontiformes	Phaethontidae	13.4	18.8
Procellariiformes	Diomedeidae	33.8	34.9
	Hydrobatidae	38.8	23.9
	Oceanitidae	55.3	23.6
	Procellariidae	52.5	28.8
	ALL	48.4	30.2
Sphenisciformes	Spheniscidae	5.15	7.75
Suliformes	Fregatidae	5.15	1.89
	Phalacrocoracidae	7.60	9.86
	Sulidae	4.69	4.74
	ALL	6.48	8.28
**all seabirds**	**all seabirds**	**31.6**	**29.5**

In our models, 95% HPD intervals of the posterior mean for most fixed effects overlapped with zero, indicating no consistent effects of any variable, except for median year, indicating that plastic load has increased over time (figure 3; supplementary material, table S5). There was strong phylogenetic signal in the model (*λ* posterior mode = 0.920, mean = 0.914; median = 0.916; 95% HPD: 0.879–0.945), and clearly identifiable phylogenetic structure in species mean weighted FO (figure 2; *λ* = 0.604) and ecological traits (supplementary material, figure S3). The main inferences of the models remained the same across all sensitivity analyses (supplementary material, tables S6–S11). Models for Charadriiformes and Procellariiformes were mostly consistent with the global model, except that carrion diets in Charadriiformes and aerial pursuit feeding in Procellariiformes were associated with lower FO (supplementary material, tables S12–13, figures S15–S16).

**Figure 3.**
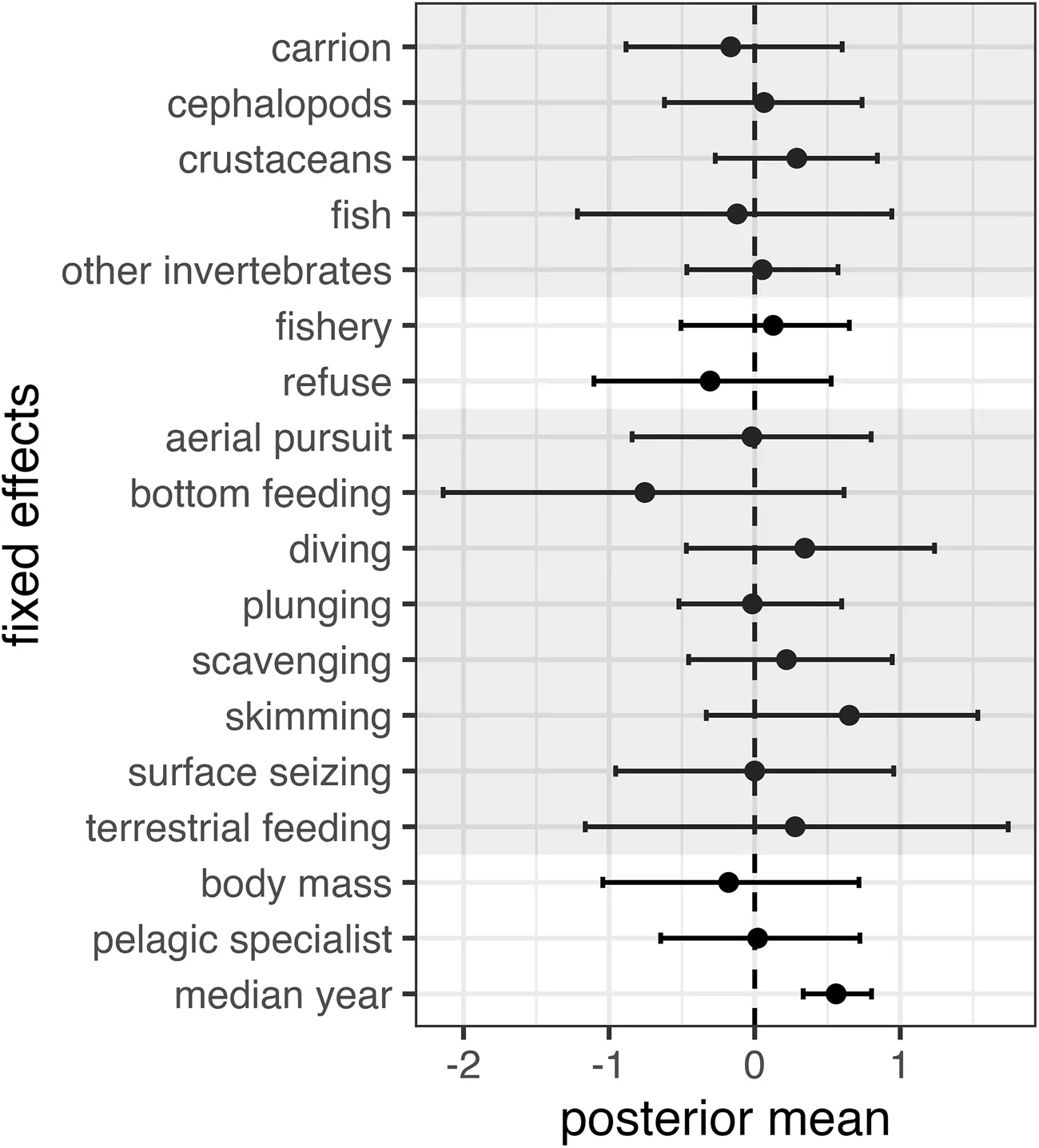
Posterior means and 95% HPD intervals for fixed effects in a phylogenetic generalized linear mixed model for the FO across 230 seabird species. Fixed effects are grouped into diet (carrion, cephalopods, crustaceans, fish and other invertebrates), anthropogenic subsidy (fishery and refuse), foraging method (aerial pursuit, bottom feeding, diving, plunging, scavenging, skimming and terrestrial feeding) and other species-level variables (body mass, pelagic specialist and median year) using shading. The dashed line indicates a posterior mean of 0.

## 4. Discussion

Our study collates the largest dataset of plastic occurrence records in seabirds to date, with data from 93 281 birds, across 240 species, recorded between 1958 and 2024. Despite this wealth of data, none of the four ecological traits in our models had a consistent effect on FO. Instead, phylogenetic relatedness was the best predictor of plastic occurrence. This does not mean ecological traits are unimportant, but instead reflects the tight correlation between seabird phylogenetic groupings and their traits that influence rates of both plastic ingestion and retention [35], including traits we have not considered here. For example, when only modelling Charadriiformes, carrion feeding is associated with lower plastic occurrence, probably because all the carrion feeders in this order also produce pellets, reducing the likelihood of detecting plastic in their guts even if they consume it. Our model also ignores more nuanced measures of the impact of plastics on seabirds, like ingested mass, which is often missing from reported data [65,66].

Most previous studies on seabirds found that diet and foraging strategy were significant predictors of plastic occurrence [24,26,30,67,68], though these studies varied in response variables, species included, and how plastic exposure was modelled. Borrelle *et al.* [69] also reported a similar result to our study, where phylogenetically conserved traits, such as body mass and foraging strategy, were not good predictors of the impact of marine threats on seabird population trends. In other studies, high plastic occurrences were found in species foraging on the surface and/or with a crustacean-dominated diet [8,24–26,70–74]. This is attributed to plastics accumulating at or near the surface of the water along with crustaceans like krill, but may also be related to differing modes of prey detection compared with diving or piscivorous species [24,75,76]. Additionally, specific dietary categories may not be as important as dietary specialization; generalists may be less selective of their prey, and therefore, have a higher risk of ingesting plastic than specialists [26,67,74,77].

The lack of effect of ecological traits in our models may alternatively be owing to trait simplification, lack of statistical power, or missing variables, including plastic retention rates in the gut. Large-scale studies, such as ours, are the only way to describe global determinants of plastic occurrence and determine whether patterns vary systematically across seabirds. Simplifying ecological traits, such as diet and foraging method, is necessary for such broad-scale evaluations. However, many ecological traits, including diet and foraging method, are influenced by age, season, breeding status, personality traits and geography [78]. Conversely, perhaps other traits are more important. Several traits influence plastic occurrence in smaller taxonomic groups, including variation in sensory systems [79], morphological traits like skull size or bill length [80], the frequency of regurgitation of indigestible material [25,67,74], and gut morphology [81,82]. Future studies should attempt to include these traits.

From a conservation point of view, our results are concerning—143 (38%) seabird species have not been studied for plastic occurrence at all, and of the 240 species with plastic occurrence records, only 98 (41%) were well-studied (at least 100 birds with at least 3 records). Pelicans (Pelecaniformes), cormorants (Phalacrocoracidae) and gulls (Laridae) require more study, aligning with broader research patterns in seabirds [83]. Our results, however, also have a positive outcome; they suggest phylogenetic position may serve as a rapid first screen for identifying species likely to accumulate plastic. For example, Procellariiformes and the families within this order frequently contain plastic [27,84–86], so it seems probable that any unstudied species within this group will have high plastic occurrence if they are exposed to plastic pollution. A logical next step would be to use this phylogenetic signal to generate predictions of plastic accumulation risk for the 143 unstudied species, prioritizing sampling efforts where predicted risk is highest.

Exposure to plastic debris is another important factor in the incidence of plastic ingestion [87]. Living in a polluted habitat can increase the likelihood of a species encountering, and therefore, ingesting plastic [23,88]. Although we collated sampling location information, it is unlikely to reflect where the bird ingested plastic. Spatially representative sampling is needed to better evaluate exposure risk, as is representativeness within populations, given that most studies report opportunistic sampling from beach-wrecked and by-caught birds [41]. As such, unhealthy or stressed birds may be oversampled, leading to biases. Many seabirds have broad ranges and/or migrate long distances [89–91], meaning we need to reduce plastics across all marine regions to reduce risks to seabirds and other wildlife.

Finally, our use of a coarse population-level metric (FO) will mask more subtle variation among taxa. Clearly, the consequences of plastic ingestion for two species with equal FO will depend on the average mass of plastic relative to their body size. Even small amounts of plastics (as low as 0.2 g) can cause significant physiological and biochemical damage [14], and effects are likely to scale with body size. Further analyses should assess the true threat of plastic pollution across all seabirds, as highlighted in a recent study that applied a risk assessment framework to the amount of plastic ingested [22]. This is exemplified by *Ardenna carneipes,* which exhibits plastic prevalence (75%–80%) comparable to other seabirds, but a single chick can contain greater than 850 plastic pieces [92]. Therefore, although this analysis is a useful step in understanding which species may be more likely to contain plastic at large scales, future work, with implementation of standardized methods, is needed.

## 5. Conclusion

Using a multi-species dataset of plastic occurrence in seabirds, we find phylogenetic relatedness is the best proxy for predicting plastic susceptibility. The long-term demographic effects of plastic on seabirds are still understudied, but it is already having disproportionate effects on rare and threatened species [24,33,90]. These effects present an additive and interacting pressure to seabird populations, already in decline as a result of by-catch, diminishing fish stocks and more frequent severe weather events with a changing climate [24]. Despite decades of evidence, the rate of policy implementation regarding the production and management of plastic waste does not reflect the urgency of the problem [1,93]. In the future, this issue will be exacerbated by increasing plastic production and subsequent marine debris accumulation [1]. Therefore, it is vital to continue the aggregation and analysis of data quantifying the extent of plastic occurrence in marine birds to mitigate its future effects. Further research should focus on sampling under-represented species, improving the standardization of methodologies, investigating species-specific morpho logical and environmental variables that may affect their risk of plastic accumulation, and targeted monitoring of the phylogenetic groups we predict to be most at risk.

## Supplementary Material



## Data Availability

All data and their descriptions/metadata are available on the NHM Data Portal at https://doi.org/10.5519/hemrjx9q. All code for data cleaning and analysis is available on GitHub at https://github.com/nhcooper123/PhyloPlastics and archived on Zenodo at https://doi.org/10.5281/zenodo.22011099. Supplementary material is available online [94].
