# Supplementary material for: Frequency of plastic occurrence varies across phylogeny but not ecology in seabirds
Source: Biol Lett. 2026 Oct 14;22(10):20260079. doi: 10.1098/rsbl.2026.0079 (PMC13682561; doi:10.1098/rsbl.2026.0079)
Supplement: Supplementary file 1 [file rsbl.2026.0079.zip › rsbl.2026.0079/rsbl20260079_si_001.pdf]

Supplementary materials from “Frequency of plastic occurrence varies across phylogeny but not ecology in seabirds”.

Stephanie Avery-Gomm, Nia Potapova, Chrissie Potter, Alexander L. Bond, Stephanie B. Borrelle, Jennifer L. Lavers, Steven J. Portugal, Hugh P. Possingham, Jennifer F. Provencher, and Natalie Cooper.

Table of contents

Table of contents......1
Details of archived datasets......2
Raw data......2
Cleaned data used in the analyses......2
Taxonomy......2
Phylogeny......2
Supplementary Methods......3
Seabird definition, taxonomy, and phylogeny preparation......3
Plastic load data: literature identification and data extraction......5
Database searches......6
Citation chasing and cross-referencing......6
Source tracking......6
Source eligibility criteria (what was included/excluded)......6
Data extraction......7
Record definition, aggregation and duplicate handling......7
Key for family-level silhouettes used in plots......10
Supplementary Results......11
Dataset coverage......11
Frequency of occurrence of plastics (FO)......19
Supplementary Results......20
MCMCglmm model outputs......20
MCMC diagnostic plots for full model......22
Sensitivity analyses......29
A. Excluding species with fewer than 10 birds in total......29
B. Excluding pelagic specialist......30
C. Excluding terrestrial feeding......31
D. Including nestbound birds......32
E. Excluding species that were added to the phylogeny (see Table S1)......33
F. Excluding studies where fewer than 10 individuals were sampled......34
Order-level results......35
A. Charadriiformes......35
B. Procellariiformes......37
Supplementary References......39

Details of archived datasets

All data are archived on the NHM Data Portal [1] at https://doi.org/10.5519/hemrjx9q. This includes the raw data and the cleaned data used in the analyses as listed below.

Raw data

- **01_sources-for-plastic-ingestion-data.** The sources from our literature searches.
- **02_plastic-ingestion-data.** The raw plastic occurrence data extracted from papers in dataset 01.
- **03_trait-data.** Species specific trait data taken from the literature.

Cleaned data used in the analyses

- **plastic-data-aggregated_2026.csv.** Aggregated data used in analyses.
- **seabird-tree-plastic-2026.nex.** Phylogeny of Claramunt et al 2025 [2] pruned to only the 230 species with plastic occurrence data.
- **full-species-list-2026.csv.** Full list of 383 seabird species along with whether or not they are found in our plastic dataset.

Taxonomy

- **seabird-list-Clements2025.csv.** List of seabird species according to Clements et al 2025 [3] taxonomy.

The full taxonomy is here: https://www.birds.cornell.edu/clementschecklist/download/ [3].

Phylogeny

- **sea-bird-tree-2026.nex.** Phylogeny of Claramunt et al. 2025 [2] pruned to seabird species only.
- **family-tree-2026.nex.** Family level phylogeny of Claramunt et al. 2025 [2] pruned to seabird families only.
- **tips-to-add.csv.** Details of 39 species missing from Claramunt et al. 2025 and how these were added to the phylogeny.

The full raw phylogeny file is here: https://raw.githubusercontent.com/evolucionario/BigBirdTree/refs/heads/main/RAGBackbone/BBtree3.tre [2].

Supplementary Methods

Seabird definition, taxonomy, and phylogeny preparation

We defined 'seabirds' as species that depend on the marine environment for at least part of their life cycle [4]. These are penguins (Sphenisciformes), albatrosses, storm-petrels, shearwaters, and petrels (Procellariiformes), frigatebirds, cormorants, gannets and boobies (Suliformes, excluding Anhingidae), tropicbirds (Phaethontiformes), pelicans (Pelecaniformes; Pelecanidae), auks (Charadriiformes; Alcidae), skuas and jaegers (Charadriiformes; Stercorariidae), phalaropes (Charadriiformes; Scolopacidae; Phalaropodini), and terns and gulls (Charadriiformes; Laridae). We also include divers (Gaviiformes), sea ducks and mergansers (Anseriformes; Anatidae; Mergini, excluding central Brazilian merganser *Mergus octosetaceus*), as these species spend the majority of the year at sea [4,5]. We followed the taxonomic treatment of Clements et al. [3], excluding eight extinct species (*Bulweria bifax, Camptorhynchus labradorius, Hydrobates macrodactylus, Mergus australis, Pinguinus impennis, Pterodroma caribbaea, Pterodroma rupinarum,* and *Urile perspicillatus*), which leaves 387 seabird species in total.

For our phylogenetically informed analyses, we used the phylogeny of Claramunt et al. [2]; specifically BBtree3) and pruned this to seabird species alone. A total of 39 seabird species were missing from the tree, primarily due to their recent discovery or elevation to species from subspecies, so we added 35 of these to the tree either as polytomies to the species they were previously defined as according to the Handbook of the Birds of the World [5], or as polytomies at the base of their Genus (see Table S1 for details). An additional four *Sterna* species (*Sterna acuticauda, Sterna aurantia, Sterna repressa*, and *Sterna virgata*) could not be added to the tree due to uncertainty about their taxonomic or phylogenetic placement, so we excluded these from our analyses leaving 383 seabird species.

Table S1. Species missing from the seabird phylogeny, along with where they were added to the phylogeny prior to the analyses, where appropriate. All information on taxonomic placements was taken from [5]. Branch lengths are either arbitrary small units, taken from an older phylogeny [6], or using dates from [2] that our seabird tree is pruned from.

Missing species	New placement	Branch length	Justification
Ardenna carneipes	*Ardenna creatopus*	1.011	Sometimes considered conspecific with *A.creatopus*. Date from [6].
Bulweria fallax	*Bulweria bulwerii*	14.83	Considered conspecific with *B.bulwerii*. Date from [6].
Diomedea sanfordi	Other *Diomedea*	0.544	Closely related to *D.epomophora*, placed outside the rest of *Diomedea*. Date from [6].
Diomedea epomophora	*Diomedea sanfordi*	0.268	Closely related to *D.sandfordi*. Date from [6].
Eudyptes chrysocome	*Eudyptes moseleyi*	0.864	Until recently considered conspecific with *E.moseleyi*. Date from [6].
Fregetta lineata	All *Fregetta*	4.860	New species, uncertain relationship to *Fregetta* spp. Used the most inclusive group containing *Fregetta* and made into a polytomy using the date from [2].

Gelochelidon macrotarsa	*Gelochelidon nilotica*	0.100	Lumped with *G.nilotica* until recently. Arbitrary date.
Gygis candida	*Gygis alba*	0.100	Lumped with *G.alba* until recently. Arbitrary date.
Larus mongolicus	*Larus argentatus*	0.100	Until recently considered conspecific with *L.argentatus*. Arbitrary date.
Larus vegae	*Larus argentatus*	0.100	Until recently considered conspecific with *L.argentatus*. Arbitrary date.
Megadyptes antipodes	All *Eudyptes*	4.052	Previously sister to all *Eudyptes*. Date from [6].
Melanitta deglandi	*Melanitta fusca*	0.100	Until recently considered conspecific with *M.fusca*. Arbitrary date.
Melanitta stejnegeri	*Melanitta deglandi*	0.100	Until recently considered conspecific with *M.deglandi*. Arbitrary date.
Microcarbo africanus	All other *Microcarbo*	10.54	Close relatives with *M.coronatus*, uncertain placement in *Microcarbo* but likely outside of the other three species.
Microcarbo coronatus	*Microcarbo africanus*	1.000	Close relatives with *M.africanus*. Arbitrary date.
Oceanites pincoyae	All *Oceanites*	13.01	New species, uncertain relationship to *Oceanites*. Used the most inclusive group containing *Oceanites* spp. and made into a polytomy using the date from [2].
Phaethon aethereus	*Phaethon lepturus*	10.79	Sister to *P.lepturus* + *P.rubricauda*. Date from [6].
Phaethon rubricauda	*Phaethon lepturus*	3.442	Sister species to *P.lepturus*. Date from [6].
Pterodroma atrata	All *Pterodroma*	25.75	*Pterodroma* hybridise so taxonomy/phylogeny is complex. All missing *Pterodroma* added as a polytomy at the base of *Pterodroma* using the date from [2].
Pterodroma defilippiana	All *Pterodroma*	25.75	*Pterodroma* hybridise so taxonomy/phylogeny is complex. All missing *Pterodroma* added as a polytomy at the base of *Pterodroma* using the date from [2].
Pterodroma gouldi	All *Pterodroma*	25.75	*Pterodroma* hybridise so taxonomy/phylogeny is complex. All missing *Pterodroma* added as a polytomy at the base of *Pterodroma* using the date from [2].
Pterodroma macroptera	All *Pterodroma*	25.75	*Pterodroma* hybridise so taxonomy/phylogeny is complex. All missing *Pterodroma* added as a polytomy at the base of *Pterodroma* using the date from [2].
Pterodroma mollis	All *Pterodroma*	25.75	*Pterodroma* hybridise so taxonomy/phylogeny is complex. All missing *Pterodroma* added as a

			polytomy at the base of *Pterodroma* using the date from [2].
Pterodroma occulta	All *Pterodroma*	25.75	*Pterodroma* hybridise so taxonomy/phylogeny is complex. All missing *Pterodroma* added as a polytomy at the base of *Pterodroma* using the date from [2].
Puffinus auricularis	All *Puffinus*	13.89	*Puffinus* taxonomy/phylogeny is in flux. All missing *Puffinus* added as a polytomy at the base of *Puffinus* using the date from [2].
Puffinus heinrothi	All *Puffinus*	13.89	*Puffinus* taxonomy/phylogeny is in flux. All missing *Puffinus* added as a polytomy at the base of *Puffinus* using the date from [2].
Puffinus lherminieri	All *Puffinus*	13.89	*Puffinus* taxonomy/phylogeny is in flux. All missing *Puffinus* added as a polytomy at the base of *Puffinus* using the date from [2].
Puffinus puffinus	All *Puffinus*	13.89	*Puffinus* taxonomy/phylogeny is in flux. All missing *Puffinus* added as a polytomy at the base of *Puffinus* using the date from [2].
Rynchops albicollis	*Rynchops niger*	6.191	Closely related to *R.niger.* Date from [6].
Rynchops flavirostris	*Rynchops niger*	0.518	Closely related to *R.niger*. Date from [6].
Sterna acuticauda	NA	NA	Uncertain phylogenetic placement. Excluded from analyses.
Sterna aurantia	NA	NA	Uncertain phylogenetic placement. Excluded from analyses.
Sterna repressa	NA	NA	Uncertain phylogenetic placement. Excluded from analyses.
Sterna virgata	NA	NA	Uncertain phylogenetic placement. Excluded from analyses.
Sternula balaenarum	*Sternula superciliaris*	0.100	Previously conspecific with *S.supercilious*. Arbitrary date.
Sternula lorata	*Hydroprogne caspia*	2.096	Previously sister to *Hydroprogne caspia*. Date from [6].
Sula brewsteri	*Sula leucogaster*	1.000	Sister to *S.leucogaster*. Arbitrary date.
Synthliboramphus hypoleucus	*Synthliboramphus scrippsi*	0.100	Conspecific with *S.scrippsi* until recently. Arbitrary date.

Plastic load data: literature identification and data extraction

The full list of plastic load sources was compiled in two stages: an initial compilation covering 1958–2017, followed by an update extending coverage through 31 Dec 2024 using the same core extraction protocol and coding rules. The workflow produces (i) a complete list of sources screened and whether they were deemed eligible to be included in the analysis (**01_sources-for-plastic-ingestion-data**), and (ii) the extracted plastic occurrence dataset used for analyses (**02_plastic-ingestion-data**).

We identified candidate sources using a combination of database searches, citation chasing, and targeted cross-referencing as described below.

Database searches

We searched Web of Science (Core Collection) for publications from 1958 to 2017, and Google Scholar for publications from 1958 to 2024, using broad and taxon-specific queries. Search strings were adapted to each platform's syntax but were conceptually equivalent. The core search terms were:

- "seabird* plastic OR debris OR pollution"

and a taxon-expanded search to capture papers that do not use the word "seabird":

- "plastic* gull OR shearwater OR albatross OR petrel OR penguin OR puffin OR auk OR guillemot OR murre* OR tern OR skua OR jaeger OR frigatebird OR booby OR gannet OR cormorant OR shag OR tropicbird OR merganser OR pelican OR phalarope OR scoter OR eider OR fulmar OR prion OR noddy OR gannet OR loon OR diver".

Citation chasing and cross-referencing

To complement keyword searches and improve coverage of older or inconsistently indexed literature, we used backward and forward citation chasing from key primary papers and reviews. In addition, we cross-referenced candidate sources against major syntheses/reviews [7–9] and seabird-related entries in LitterBase [10], retrieving and screening any additional primary sources identified through these routes.

Source tracking

All publications identified during screening (including those later excluded) were downloaded/recorded in **01_sources-for-plastic-ingestion-data**. Only studies that met inclusion criteria were extracted into **02_plastic-ingestion-data.**

Source eligibility criteria (what was included/excluded)

We included sources involving study of wild (non-captive) seabird species as defined in the Seabird definition, taxonomy, and phylogeny preparation section. We included studies reporting plastic ingestion of any plastic type and any size class (micro <5 mm, meso 5–20 mm, macro >20 mm). Both plastic-focused studies and studies where plastic was reported incidentally (e.g., diet/stomach-content studies) were eligible, provided the source reported (or allowed derivation of) the number examined and the number with plastic. We included any sampling approach capable of detecting ingested plastic, including necropsy, lavage, bolus/regurgitation-based approaches, scat, and related methods (coded as described below). In a small number of cases where an otherwise eligible study did not report extractable counts (n examined and/or n with plastic) in the publication, we contacted lead authors to obtain the underlying data. This was done for three publications [11–13], enabling inclusion using the same extraction and coding rules as for published data.

We excluded sources on studies for species outside our seabird definition (e.g. extinct species, freshwater-only species), captive populations, and experimental exposures. We excluded sources reporting on entanglement only (with no ingestion data), and studies that pooled plastic with non-plastic marine debris in a way that prevented extraction of plastic-specific counts (e.g., combined totals included hooks, glass, rubber). Sources that did not report, and did not allow derivation of, both the number of birds examined and the number with plastic were excluded. Finally, we removed duplicate or secondary reporting of the same underlying dataset when this was clearly identified by the authors (see Duplicate handling). Duplicate data from secondary research was removed as long as it was specified as such by the author.

Data extraction

For each eligible publication, we extracted the species, the number of individuals examined (n total) and the number with plastic (n plastic), year(s) of sampling, sampling method (bolus, brushing, lavage, necropsy, regurgitation or scat), and ocean basin (Arctic, Indian, North Atlantic, North Pacific, South Atlantic, South Pacific, or Southern Ocean). Where finer-scale location information was reported, it was retained in the dataset, but ocean basin was the standardized spatial category used in the models.

We recorded the sampling year(s) reported for each record. When sampling spanned multiple years within a record, we derived a single “median year of data collection” value for modelling (midpoint of a reported year range or the median of discrete years, as applicable).

We categorised the reported age class of sampled individuals as adult, subadult, adult/subadult, nest-bound (chicks or fledglings), or mixed. If birds were collected at sea and age class was not reported, we coded age as adult/subadult (i.e., not nest-bound). When multiple age classes were pooled or age class could not be discerned, we coded age as mixed. For analyses, age class was then aggregated into free-flying, nest-bound, or mixed; nest-bound records were excluded from the main models because those individuals reflect parental foraging rather than individual exposure/selection.

We did not extract plastic type/shape/colour or plastic loads (mass or item counts) because these metrics were too inconsistently reported across sources to support a standardized synthesis at this scale.

Record definition, aggregation and duplicate handling

Within each source, we extracted data as separate records when the authors reported clearly distinct sampling units (i.e., unique combinations of source, year(s), site/region where available, species, age class, and sampling method). Where studies did not provide enough detail to separate subgroups reliably, we extracted the data at the level reported.

To avoid double counting, we ensured the analysis dataset contained only one entry for each sampling unit. When sources reported multiple overlapping summaries, we retained the most disaggregated (primary) data available and excluded redundant aggregations. We also removed duplicate/secondary reporting of the same underlying dataset when this was explicitly identified by the authors as previously published.

Frequency of occurrence (FO) was calculated for each record as FO = n plastic / n examined. For species-level summaries (e.g., figures), we calculated weighted mean FO across records for each species using n examined as weights.

Table S2: Correlation matrix of variables in the full models. Correlations > 0.5 are in bold.

term	freeflying	body_ mass_2sd	carrion	fish	cephalopods	crustaceans	other_ inverts	as_fishery	as_refuse	pelagic_ specialist	aerial_ pursuit	bottom_ feeding	diving	plunging	scavenging	skimming	surface_ seizing	terrestrial_ feeding	year
freeflying	NA	-0.047	-0.084	0.006	0.128	0.058	-0.031	-0.031	-0.114	0.171	-0.041	-0.084	-0.002	0.058	-0.047	0.138	0.053	-0.148	-0.022
body_ mass_2sd	-0.047	NA	0.017	0.039	0.085	-0.002	-0.152	0.093	-0.043	0.018	-0.049	0.032	0.058	-0.035	0.152	-0.135	-0.126	0.029	-0.026
carrion	-0.084	0.017	NA	0.008	-0.135	0.018	0.276	0.362	0.378	-0.259	0.202	-0.007	-0.109	0.108	0.473	-0.077	0.155	**0.562**	0.056
fish	0.006	0.039	0.008	NA	0.130	-0.068	-0.096	0.176	0.054	0.040	0.050	-0.066	-0.086	0.178	0.123	0.072	0.129	-0.011	0.037
cephalopods	0.128	0.085	-0.135	0.130	NA	0.137	-0.195	0.091	-0.360	0.494	-0.057	-0.206	-0.142	0.185	0.050	0.139	0.305	-0.353	0.055
crustaceans	0.058	-0.002	0.018	-0.068	0.137	NA	0.200	0.135	-0.017	0.070	-0.171	0.106	0.038	-0.068	-0.121	0.143	-0.001	-0.093	0.008
other_ inverts	-0.031	-0.152	0.276	-0.096	-0.195	0.200	NA	0.191	0.187	-0.262	-0.025	0.150	0.014	-0.055	-0.052	-0.195	-0.039	0.149	0.004
as_fishery	-0.031	0.093	0.362	0.176	0.091	0.135	0.191	NA	0.208	0.000	0.071	-0.211	-0.285	0.365	0.461	-0.018	0.396	0.230	0.086
as_refuse	-0.114	-0.043	0.378	0.054	-0.360	-0.017	0.187	0.208	NA	-0.257	0.099	-0.056	-0.188	-0.039	0.284	-0.081	0.087	**0.677**	-0.022
pelagic_ specialist	0.171	0.018	-0.259	0.040	0.494	0.070	-0.262	0.000	-0.257	NA	-0.016	-0.424	-0.026	0.116	-0.009	0.078	0.055	-0.344	0.021
aerial_ pursuit	-0.041	-0.049	0.202	0.050	-0.057	-0.171	-0.025	0.071	0.099	-0.016	NA	-0.061	-0.172	-0.019	0.150	-0.082	-0.097	0.230	0.051
bottom_ feeding	-0.084	0.032	-0.007	-0.066	-0.206	0.106	0.150	-0.211	-0.056	-0.424	-0.061	NA	0.333	-0.289	-0.202	-0.080	-0.350	-0.087	-0.034
diving	-0.002	0.058	-0.109	-0.086	-0.142	0.038	0.014	-0.285	-0.188	-0.026	-0.172	0.333	NA	-0.232	-0.237	-0.248	**-0.532**	-0.153	0.031
plunging	0.058	-0.035	0.108	0.178	0.185	-0.068	-0.055	0.365	-0.039	0.116	-0.019	-0.289	-0.232	NA	0.338	0.258	0.480	0.067	0.032
scavenging	-0.047	0.152	0.473	0.123	0.050	-0.121	-0.052	0.461	0.284	-0.009	0.150	-0.202	-0.237	0.338	NA	0.038	0.335	0.415	0.075
skimming	0.138	-0.135	-0.077	0.072	0.139	0.143	-0.195	-0.018	-0.081	0.078	-0.082	-0.080	-0.248	0.258	0.038	NA	0.183	-0.122	0.036
surface_ seizing	0.053	-0.126	0.155	0.129	0.305	-0.001	-0.039	0.396	0.087	0.055	-0.097	-0.350	**-0.532**	0.480	0.335	0.183	NA	0.097	0.013
terrestrial_ feeding	-0.148	0.029	**0.562**	-0.011	-0.353	-0.093	0.149	0.230	**0.677**	-0.344	0.230	-0.087	-0.153	0.067	0.415	-0.122	0.097	NA	0.045
year	-0.022	-0.026	0.056	0.037	0.055	0.008	0.004	0.086	-0.022	0.021	0.051	-0.034	0.031	0.032	0.075	0.036	0.013	0.045	NA

Key for family-level silhouettes used in plots

Order	Family	Silhouette	Order	Family	Silhouette
Anseriformes	Anatidae	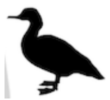	Procellariiformes	Diomedeidae	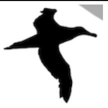
Charadriiformes	Alcidae	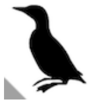	Procellariiformes	Hydrobatidae	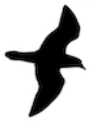
Charadriiformes	Laridae	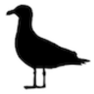	Procellariiformes	Oceanitidae	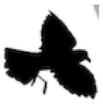
Charadriiformes	Scolopacidae	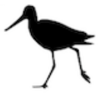	Procellariiformes	Procellariidae	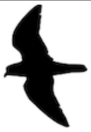
Charadriiformes	Stercorariidae	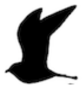	Sphenisciformes	Spheniscidae	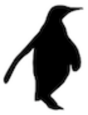
Gaviiformes	Gaviidae	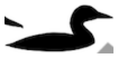	Suliformes	Fregatidae	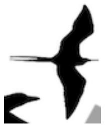
Pelecaniformes	Pelecanidae	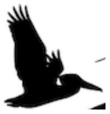	Suliformes	Phalacrocoracidae	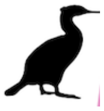
Phaethontiformes	Phaethontidae	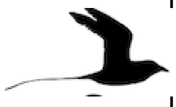	Suliformes	Sulidae	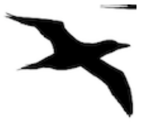

Supplementary Results

Dataset coverage

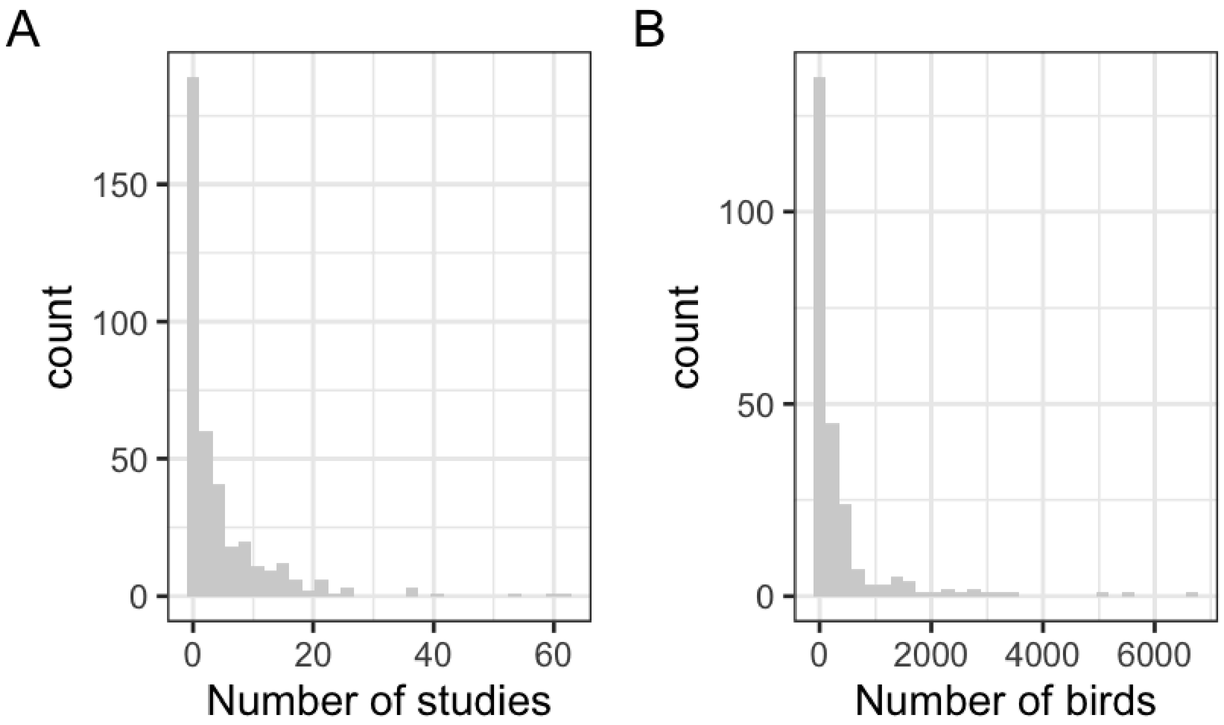

Figure S1: Histogram of the distribution of number of studies (A) and number of birds (B) for each species in the dataset.

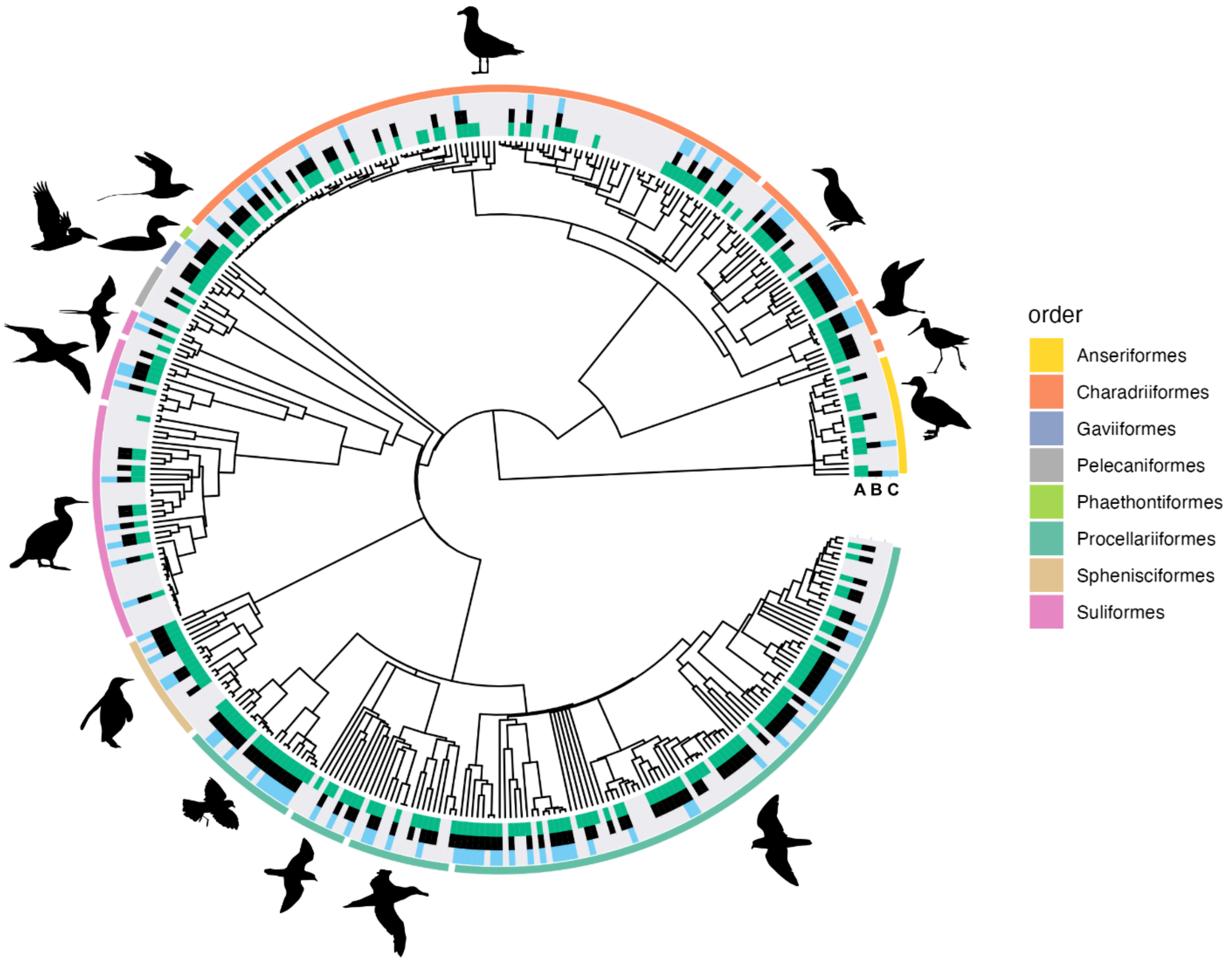

Figure S2: Summary of plastic ingestion research for the 383 seabird species defined by [3] and in the phylogeny of [2]. A) Species that have been studied in plastic occurrence research; B) species that have been recorded with plastic; C) species that have been well-studied in plastic occurrence research, i.e. ≥ 100 birds sampled across ≥ 3 records. Clade labels are coloured by order. Families are indicated by silhouettes (all from phylopic.org, CC0 1.0 license; key above). To aid readability, species names are omitted.

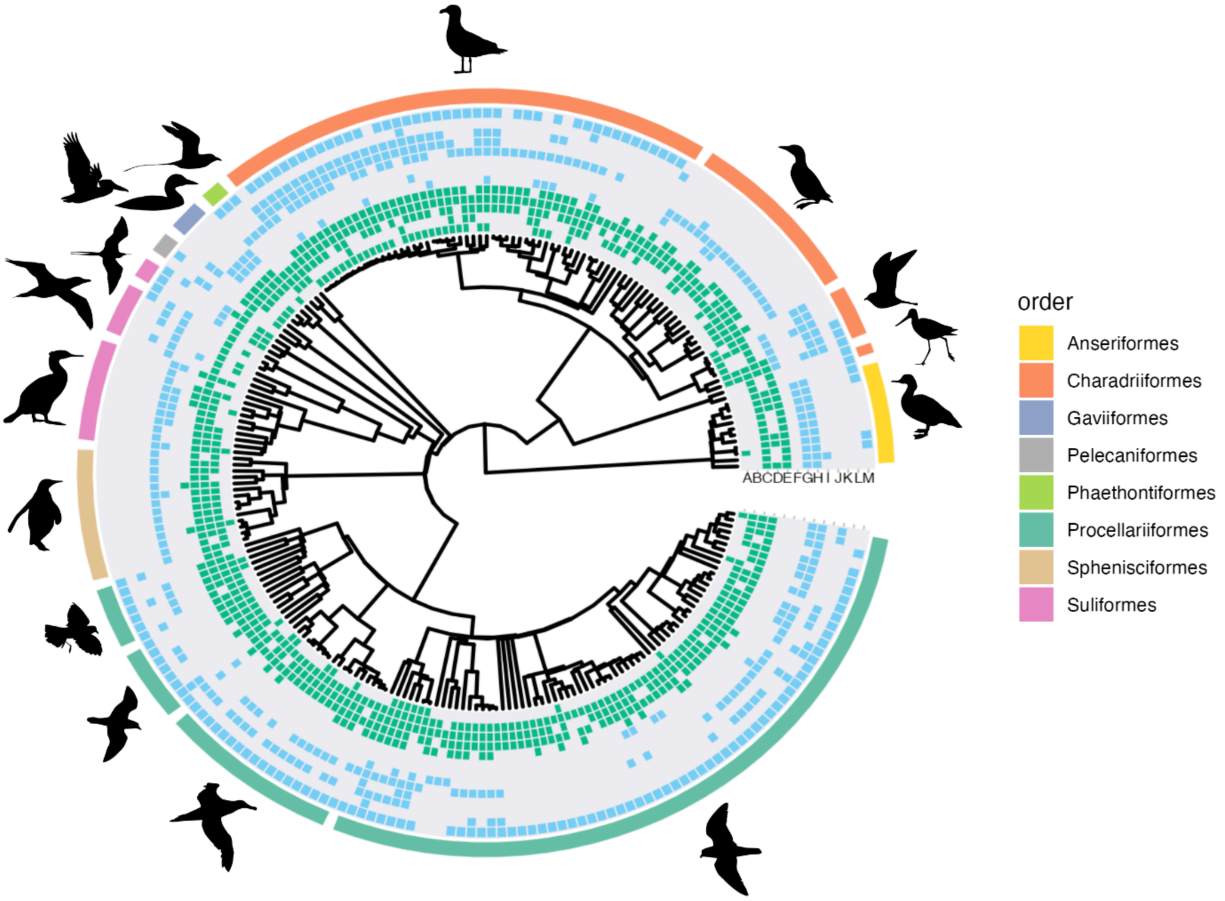

Figure S3. Phylogenetic tree of the 230 seabird species in our analyses annotated with diet and foraging method. A-E shows whether each item forms part of the species diet. F-M shows whether a species uses a particular foraging method. A) carrion/non fish vertebrates; B) cephalopods; C) crustaceans; D) fish; E) other invertebrates. F) aerial pursuit; G) bottom feeding; H) diving; I) plunging; J) terrestrial feeding; K) scavenging; L) skimming; and M) surface seizing. Clade labels are coloured by order. Families are indicated by silhouettes (all from phylopic.org, CC0 1.0 license; key above). To aid readability, species names are omitted.

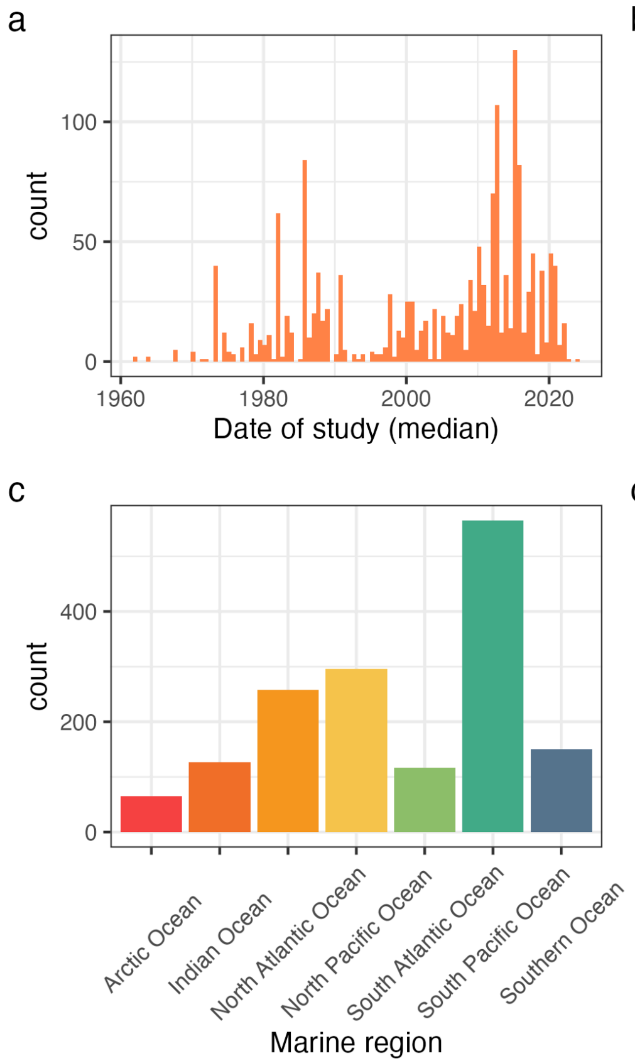

Figure S4. Distribution of study-level variables for plastic occurrence studies. a) median date of study (years); b) age class of birds in the study; c) ocean basin; and d) sampling method. Note that these plots include only studies used in the final analyses.

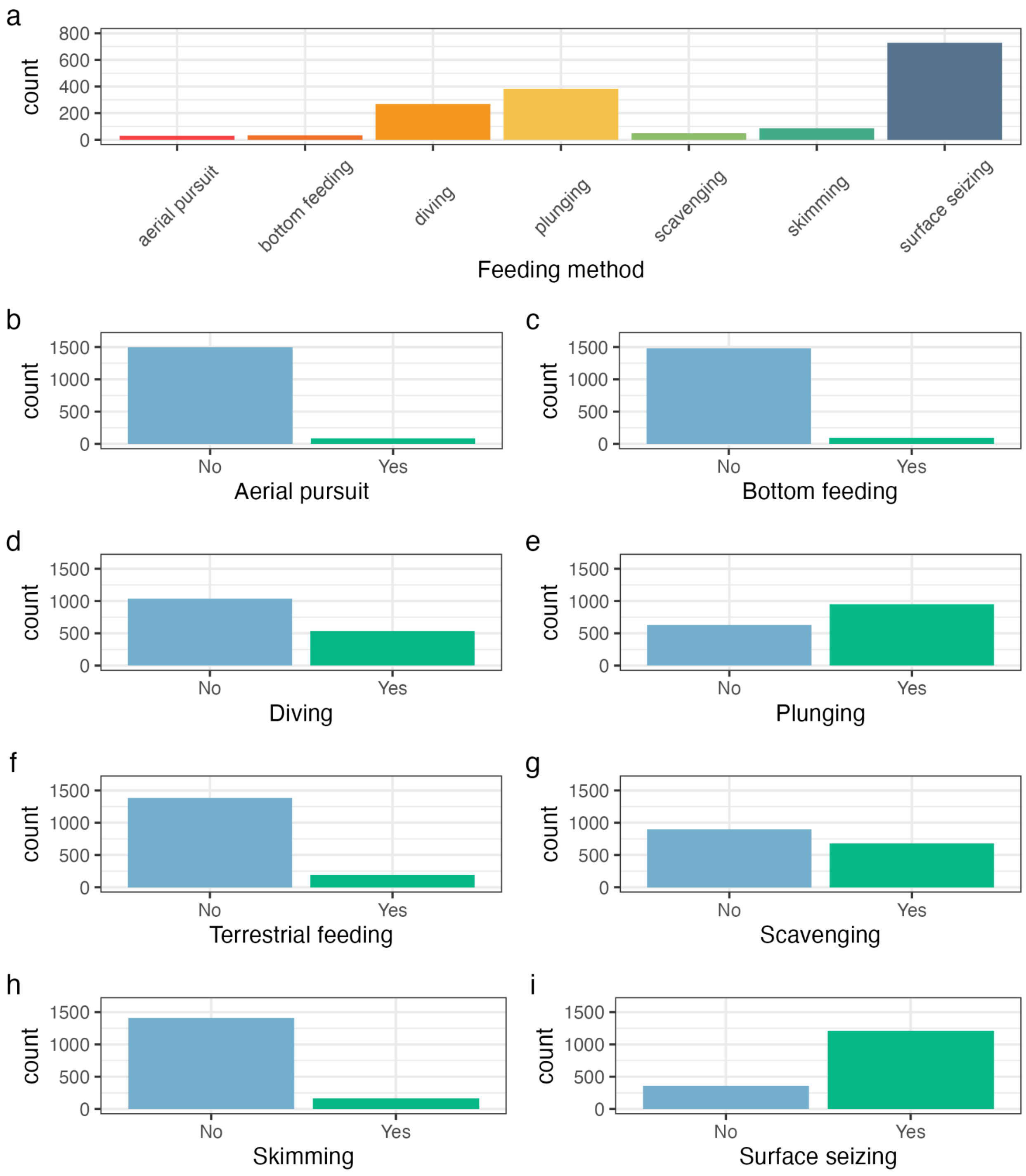

Figure S5. Distribution of foraging methods across the plastic occurrence studies. Numbers show the number of studies containing a species with each foraging method, thus some species will be counted more than once if they appear in more than one study. Each species may have multiple foraging methods. a) primary foraging method; b) aerial pursuit; c) bottom feeding; d) diving; e) plunging; f) terrestrial feeding; g) scavenging; h) skimming; and i) surface seizing. Note that these plots only include studies used in the final analyses.

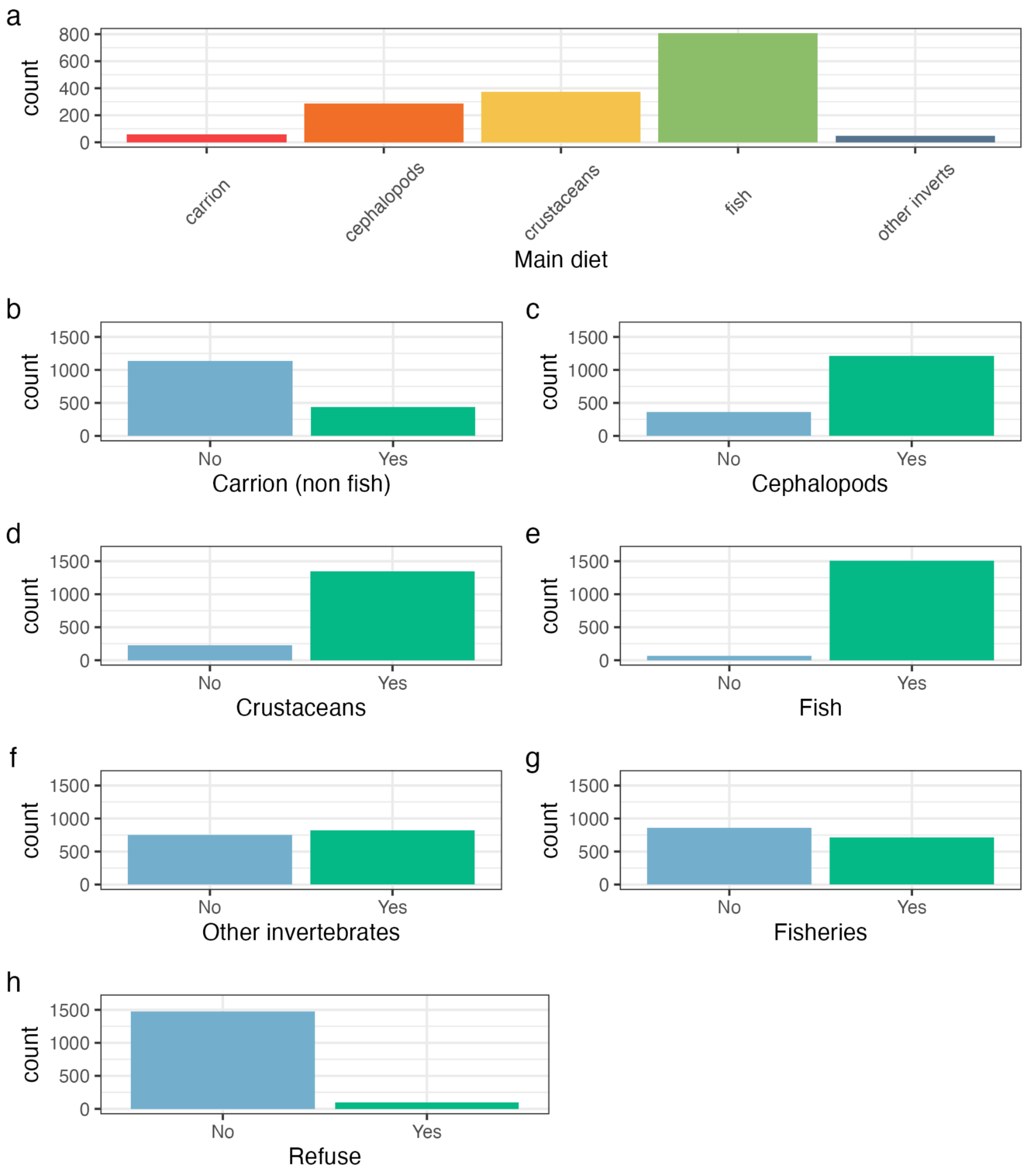

Figure S6. Distribution of diets across the plastic occurrence studies. Numbers show the number of studies containing a species with each diet, thus some species will be counted more than once if they appear in more than one study. Each species may have multiple diets. a) main diet; b) carrion/vertebrates other than fish; c) cephalopods; d) crustaceans; e) fish; f) other invertebrates (i.e. not cephalopods or crustaceans); g) anthropogenic subsidy from fisheries discards; and h) anthropogenic subsidy from refuse. Note that these plots only include studies used in the final analyses.

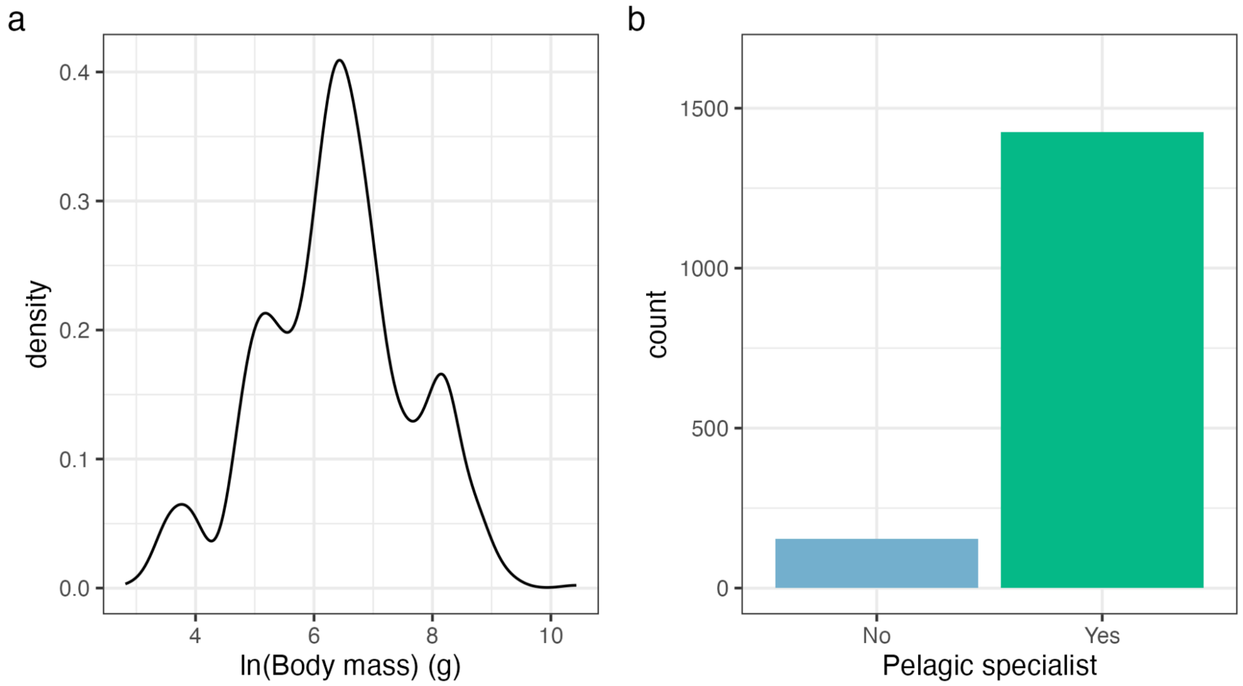

Figure S7. Distribution of body sizes and pelagic specialism across the plastic occurrence studies. Numbers show the number of studies containing a species with each body mass or pelagic specialism, thus some species will be counted more than once if they appear in more than one study. a) natural log-transformed body mass (g); and b) pelagic specialist. Note that these plots only include studies used in the final analyses.

*Table S3. Dataset coverage across taxonomic groups. Numbers in the table indicate the total number of species, or the total number of plastic occurrence studies, in each Order and Family of seabirds. Columns show (1) the number of species in the taxonomy of [3] and the phylogeny of [2]; (2) the number of species included in studies that are part of our plastic occurrence dataset; (3) the % coverage of species in found in our plastic occurrence dataset; (4) the number of species included in our analyses, i.e. after excluding species/studies with incomplete life history or study-level data; (5) the total number of studies in our plastic occurrence dataset; (6) the total number of studies in our plastic occurrence dataset that are included in our analyses, i.e. after excluding species/studies with incomplete life history or study-level data; (7) the total number of individual birds in our plastic occurrence dataset; (8) the total number of individual birds in our plastic occurrence dataset that are included in our analyses, i.e. after excluding species/studies with incomplete life history or study-level data. *Four species of Sterna were removed because they could not be placed in the phylogeny (see Table S1).*

		**Species**				**Studies**		**Individual birds**	
**Order**	**Family**	**Total**	**Dataset**	**% coverage**	**Models**	**Total**	**Models**	**Total**	**Models**
Anseriformes	Anatidae	20	13	65	11	40	28	1,931	1,809
Charadriiformes	Alcidae	24	18	75	18	164	159	8,966	8,661
	Laridae	97*	49	48.5	48	257	226	12,691	11,250
	Scolopacidae	3	2	66.7	2	9	7	48	26
	Stercorariidae	7	6	85.7	6	30	29	7,561	7,558
	ALL	*131*	*75*	*57.3*	*74*	*460*	*421*	*29,266*	*27,495*
Gaviiformes	Gaviidae	5	5	100	4	13	10	82	71
Pelecaniformes	Pelecanidae	8	3	37.5	3	4	3	45	31
Phaethontiformes	Phaethontidae	3	3	100	3	19	15	636	468
Procellariiformes	Diomedeidae	20	19	95	18	194	158	7,089	4,842
	Hydrobatidae	17	10	58.8	8	58	44	4,210	3,698
	Oceanitidae	10	7	70	7	41	38	1,861	1,845
	Procellariidae	96	69	71.1	68	772	718	32,107	28,749
	ALL	*143*	*105*	*73.4*	*101*	*1065*	*958*	*45,267*	*39,134*
Sphenisciformes	Spheniscidae	19	14	73.7	14	59	53	8,326	8,264
Suliformes	Fregatidae	5	3	60	3	10	8	485	439
	Phalacrocoracidae	33	13	39.4	11	47	39	4,687	2,325
	Sulidae	11	6	54.5	6	46	39	2,556	2,370
	ALL	*49*	*22*	*44.9*	*20*	*103*	*86*	*7,728*	*5,134*
**TOTAL**		**383**	**240**	**62.7**	**230**	**1,763**	**1,574**	**93,281**	**82,406**

Frequency of occurrence of plastics (FO)

Table S4. Frequency of occurrence of plastic (FO) for the 37 species in our dataset with FO >= 50%. n is the number of birds studied.

Order	Family	Species	n	FO (%)
Charadriiformes	Laridae	*Leucophaeus atricilla*	13	100
Pelecaniformes	Pelecanidae	*Pelecanus occidentalis*	26	100
Procellariiformes	Procellariidae	*Pseudobulweria aterrima*	1	100
Suliformes	Phalacrocoracidae	*Phalacrocorax sulcirostris*	1	100
Procellariiformes	Hydrobatidae	*Hydrobates monorhis*	305	96.4
Procellariiformes	Procellariidae	*Ardenna bulleri*	122	95.9
Procellariiformes	Hydrobatidae	*Hydrobates furcatus*	41	92.7
Procellariiformes	Diomedeidae	*Phoebastria immutabilis*	1,439	89.4
Charadriiformes	Laridae	*Larus pacificus*	481	86.7
Procellariiformes	Procellariidae	*Pterodroma sandwichensis*	7	85.7
Procellariiformes	Hydrobatidae	*Hydrobates tristrami*	221	85.5
Procellariiformes	Procellariidae	*Fulmarus glacialis*	5,556	83.8
Procellariiformes	Procellariidae	*Ardenna tenuirostris*	3,323	82.4
Procellariiformes	Procellariidae	*Halobaena caerulea*	166	79.5
Procellariiformes	Procellariidae	*Calonectris borealis*	2,135	79.3
Procellariiformes	Procellariidae	*Puffinus bailloni*	56	78.6
Charadriiformes	Laridae	*Larus glaucoides*	21	76.2
Procellariiformes	Procellariidae	*Ardenna gravis*	1,300	73.8
Procellariiformes	Diomedeidae	*Phoebastria nigripes*	471	73.7
Procellariiformes	Procellariidae	*Puffinus yelkouan*	31	71.0
Procellariiformes	Procellariidae	*Puffinus mauretanicus*	47	70.2
Procellariiformes	Oceanitidae	*Pelagodroma marina*	1,383	69.0
Procellariiformes	Procellariidae	*Pterodroma baraui*	208	68.3
Procellariiformes	Procellariidae	*Pterodroma brevipes*	3	66.7
Procellariiformes	Procellariidae	*Pterodroma longirostris*	72	66.7
Procellariiformes	Procellariidae	*Ardenna carneipes*	2,734	65.17
Procellariiformes	Diomedeidae	*Phoebastria albatrus*	17	64.77
Suliformes	Phalacrocoracidae	*Nannopterum auritum*	74	60.87
Charadriiformes	Alcidae	*Aethia psittacula*	468	60.7
Charadriiformes	Laridae	*Larus smithsonianus*	90	60.0
Procellariiformes	Procellariidae	*Ardenna grisea*	1,887	58.7
Phaethontiformes	Phaethontidae	*Phaethon aethereus*	97	55.7
Procellariiformes	Procellariidae	*Pachyptila desolata*	164	53.0
Charadriiformes	Laridae	*Larus marinus*	160	50.0
Procellariiformes	Procellariidae	*Ardenna creatopus*	6	50.0
Procellariiformes	Procellariidae	*Pterodroma leucoptera*	32	50.0
Procellariiformes	Procellariidae	*Pterodroma phaeopygia*	2	50.0

Supplementary Results

MCMCglmm model outputs

Table S5. Results from a phylogenetic generalised linear mixed model for the frequency of occurrence of plastic (FO) across 230 seabird species with behavioural and life-history traits. HPD = highest posterior density; ESS = effective sample size. Posterior mode of lambda (λ) = 0.920. Posterior mean of lambda (λ) = 0.914. Posterior median of lambda (λ) = 0.916. 95% HPD of lambda (λ) = 0.882 - 0.947.

**Fixed effects**	**posterior mean**	**lower 95% HPD**	**upper 95% HPD**	**ESS**
Median year of study	0.559	0.334	0.803	723
**Diet**				
Carrion	-0.165	-0.884	0.601	921
Cephalopods	0.065	-0.620	0.738	950
Crustaceans	0.290	-0.271	0.843	950
Fish	-0.120	-1.217	0.942	950
Other invertebrates	0.052	-0.466	0.572	950
**Anthropogenic subsidy**				
Fisheries	0.127	-0.506	0.650	950
Refuse	-0.306	-1.104	0.526	1079
**Foraging method**				
Aerial pursuit	-0.019	-0.842	0.800	950
Bottom feeding	-0.755	-2.140	0.614	1169
Diving	0.344	-0.469	1.235	950
Plunging	-0.016	-0.521	0.598	950
Scavenging	0.219	-0.454	0.945	950
Skimming	0.650	-0.333	1.533	950
Surface seizing	0.000	-0.955	0.955	1129
Terrestrial feeding	0.278	-1.164	1.742	950
**Other species-level variables**				
Body mass (scaled)	-0.179	-1.043	0.716	796
Pelagic specialist	0.020	-0.646	0.723	950
**Random effects**				
Free-flying	9.391	0.002	12.832	631
Ocean basin	0.350	0.021	0.957	950
Source	3.322	2.467	4.305	950
Species	13.623	8.788	18.62	950

MCMC diagnostic plots for full model

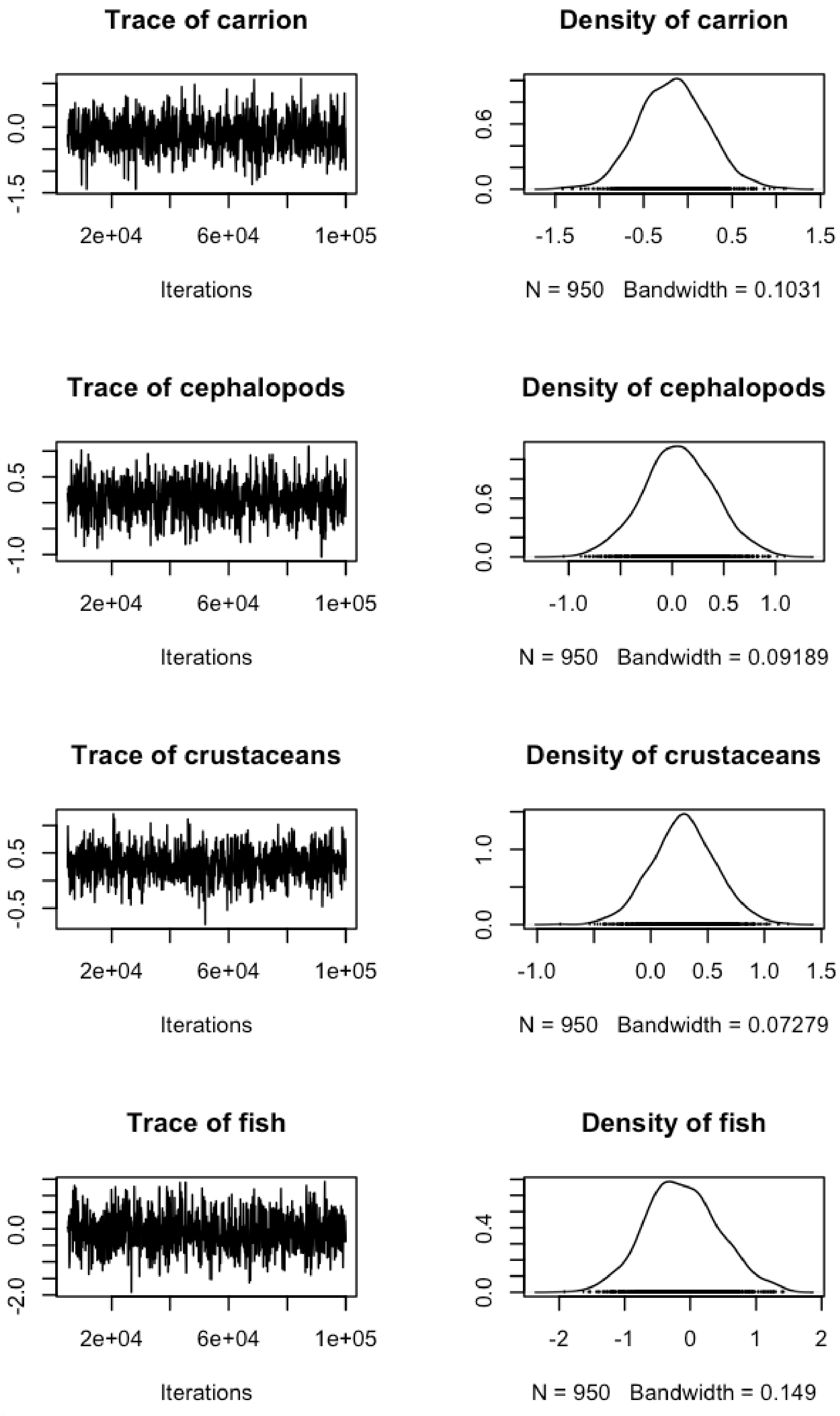

Figure S8: Traceplots and posterior densities for the variables carrion, cephalopods, crustaceans and fish from the full MCMCglmm model.

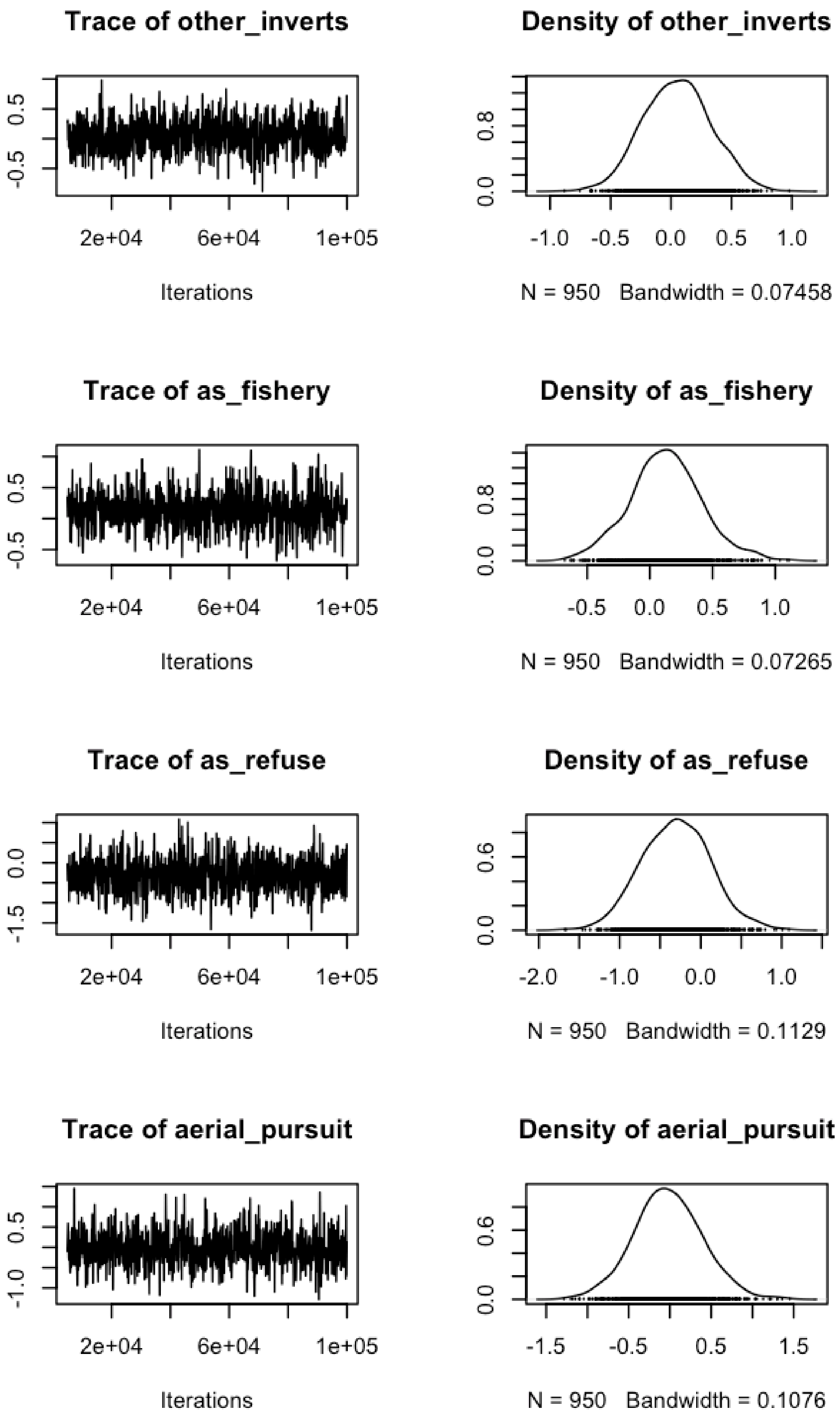

Figure S9: Traceplots and posterior densities for the variables other inverts, anthropogenic subsidy: refuse, anthropogenic subsidy: fisheries and aerial pursuit from the full MCMCglmm model.

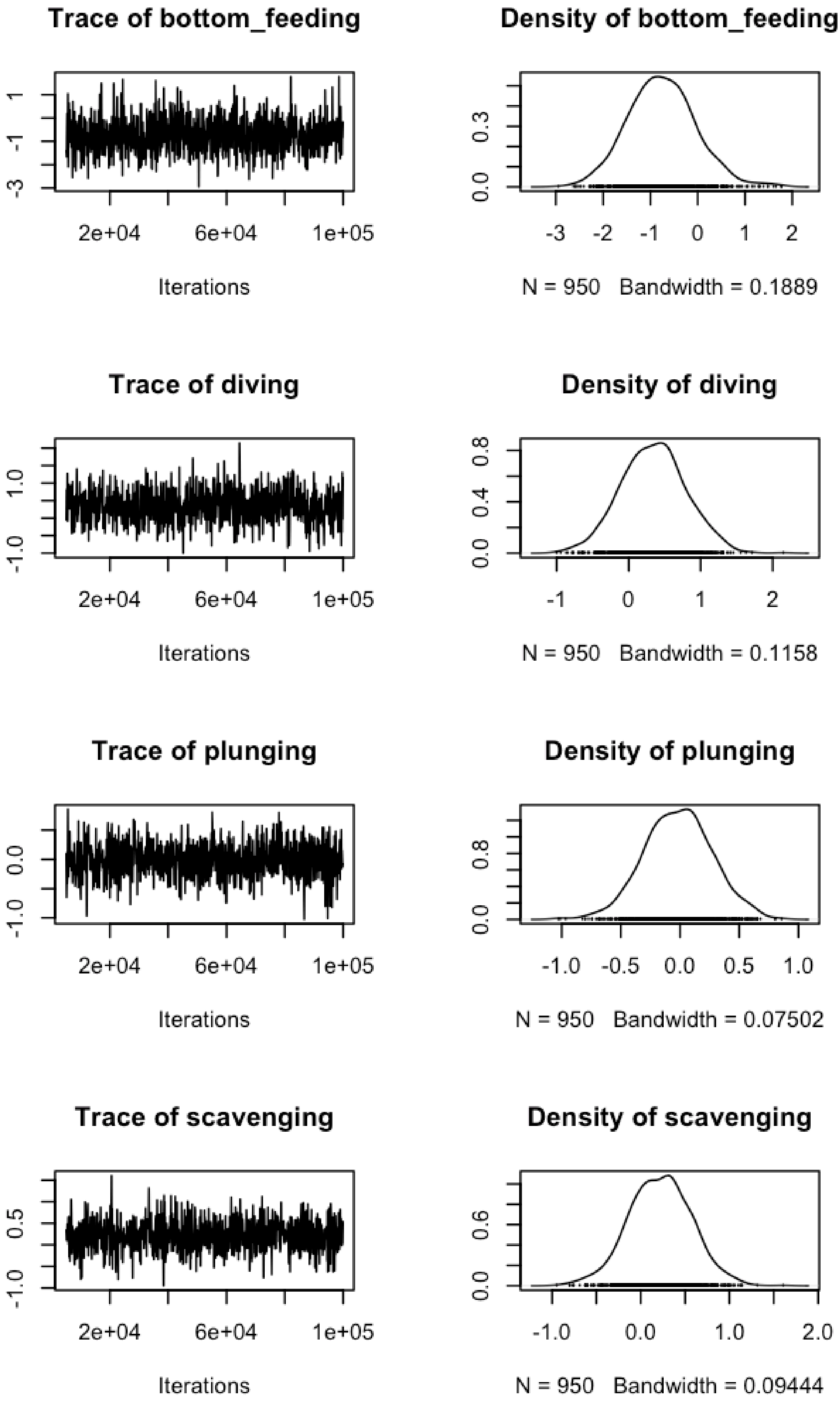

Figure S10: Traceplots and posterior densities for the variables bottom feeding, diving, plunging and scavenging from the full MCMCglmm model.

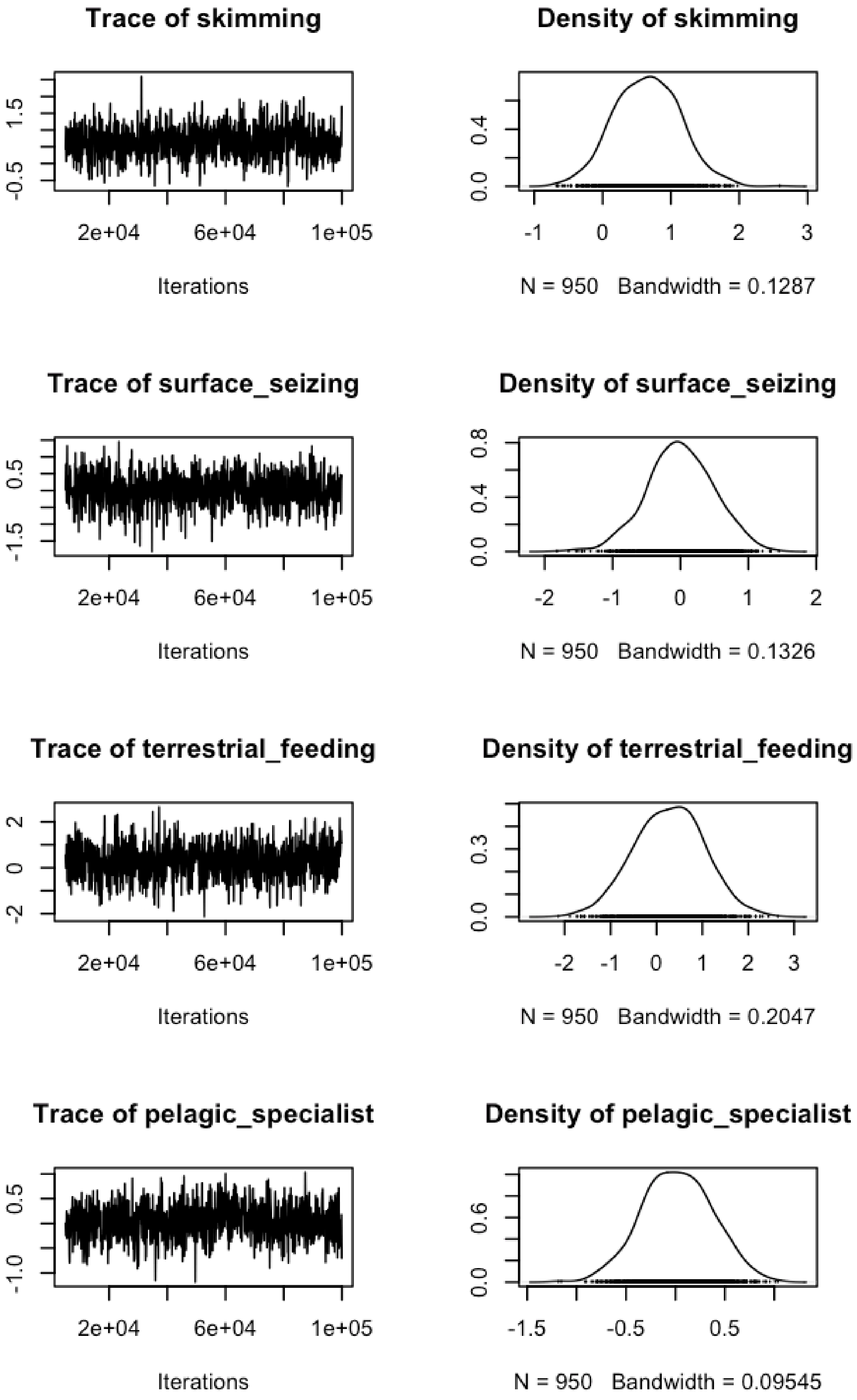

Figure S11: Traceplots and posterior densities for the variables skimming, surface seizing, terrestrial feeding and pelagic specialist from the full MCMCglmm model.

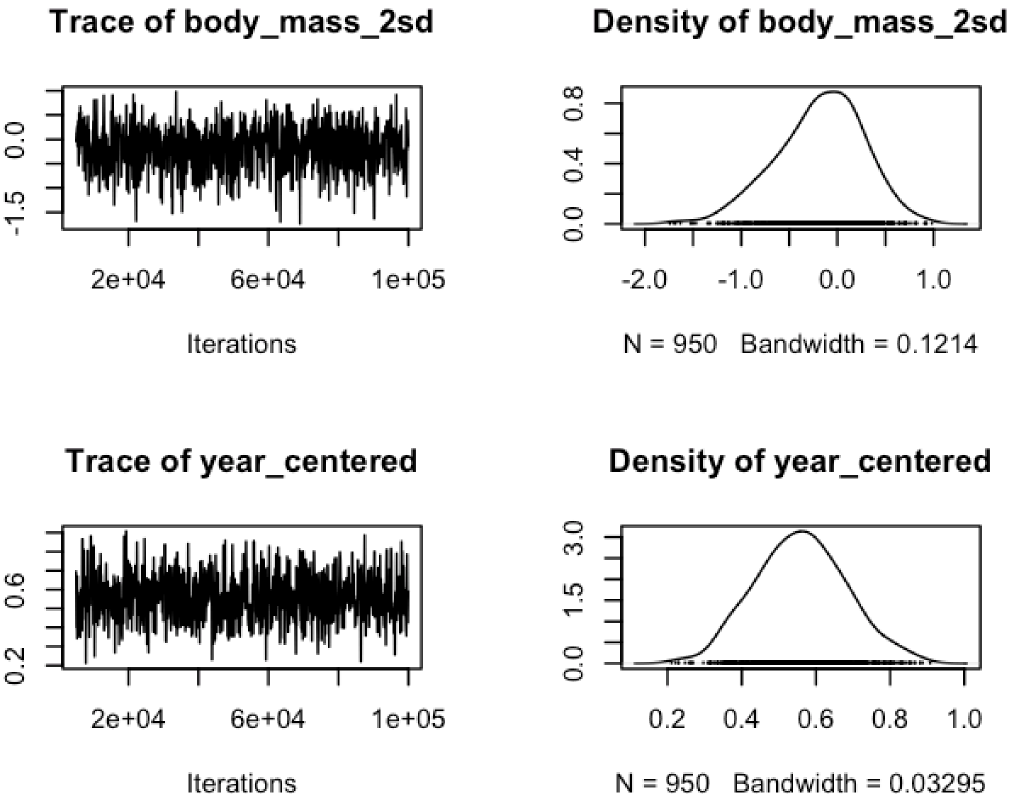

Figure S12: Traceplots and posterior densities for the variables body mass and year of study from the full MCMCglmm model.

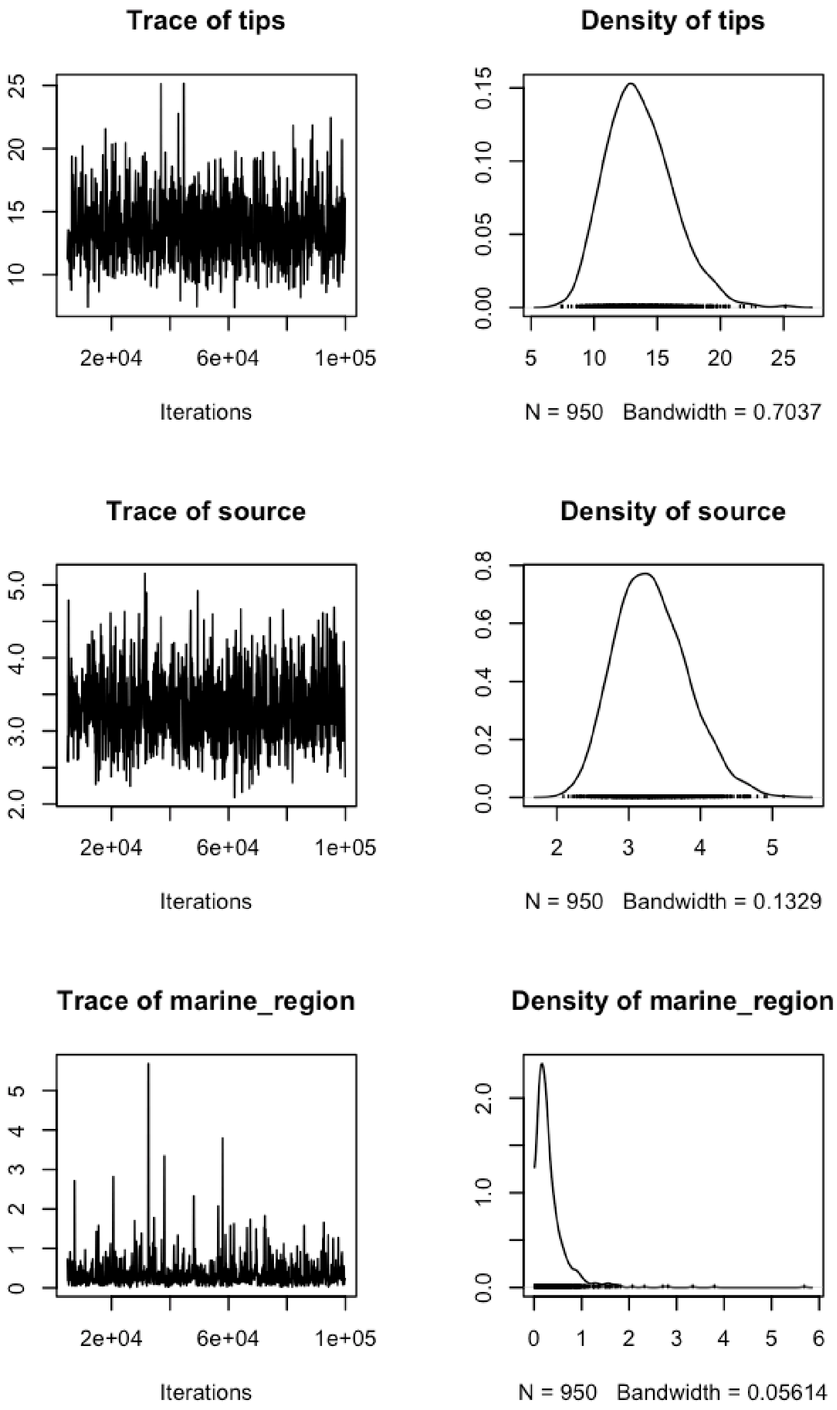

Figure S13: Traceplots and posterior densities for the random effects tips, source and marine region from the full MCMCglmm model.

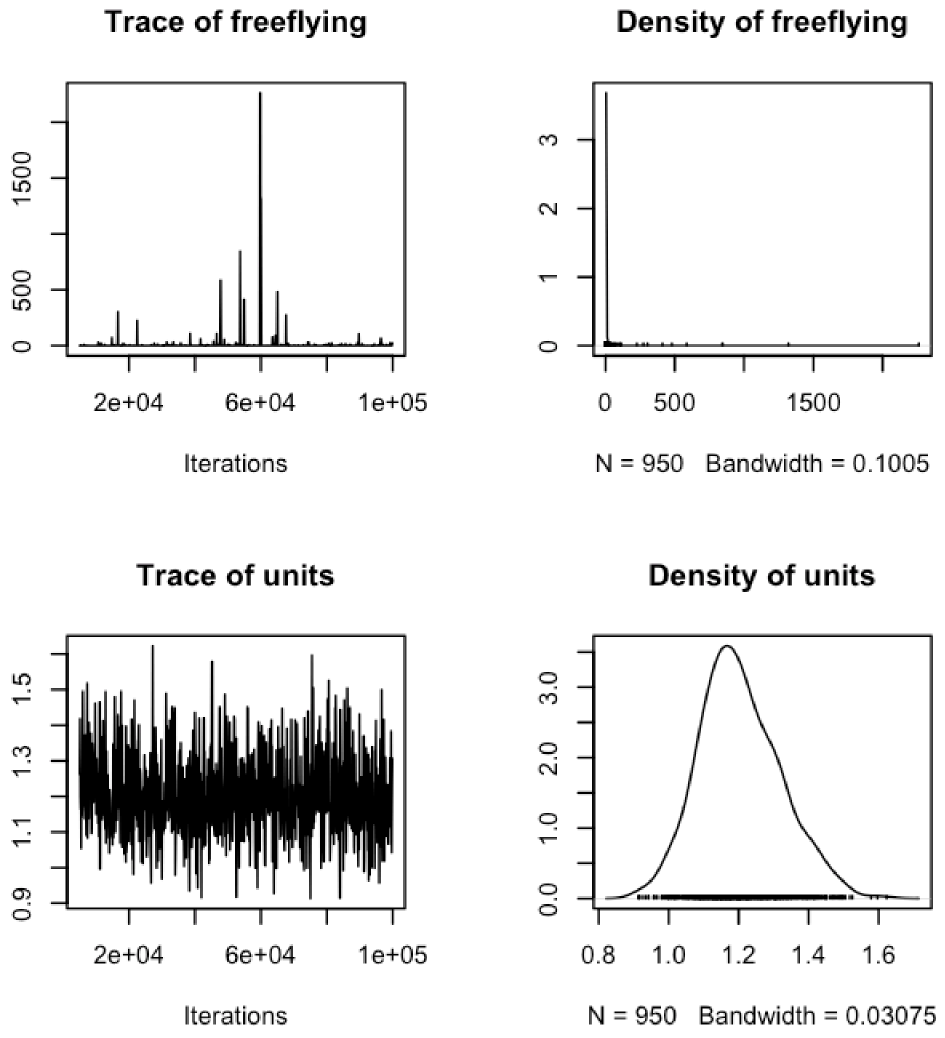

Figure S14: Traceplots and posterior densities for the random effect freeflying and for the units from the full MCMCglmm model.

Sensitivity analyses

A. Excluding species with fewer than 10 birds in total

Table S6. Results from a phylogenetic generalised linear mixed model for the frequency of occurrence of plastic (FO) across 175 seabird species with behavioural and life-history traits excluding the pelagic specialist variable. HPD = highest posterior density; ESS = effective sample size. Posterior mode of lambda (λ) = 0.928 (95% HPD: 0.888 - 0.957).

**Fixed effects**	**posterior mean**	**lower 95% HPD**	**upper 95% HPD**	**ESS**
Median year of study	0.631	0.378	0.906	1052
**Diet**				
Carrion	0.099	-0.774	1.026	950
Cephalopods	-0.106	-1.048	0.685	817
Crustaceans	0.331	-0.343	1.005	950
Fish	0.815	-0.694	2.243	950
Other invertebrates	0.176	-0.475	0.791	950
**Anthropogenic subsidy**				
Fisheries	0.091	-0.529	0.763	950
Refuse	-0.676	-1.705	0.285	950
**Foraging method**				
Aerial pursuit	0.036	-0.902	0.986	950
Bottom feeding	-0.652	-2.192	0.797	1001
Diving	0.517	-0.613	1.376	1143
Plunging	-0.038	-0.727	0.685	1076
Scavenging	0.753	-0.126	1.506	950
Skimming	0.533	-0.513	1.491	1093
Surface seizing	-0.294	-1.626	0.911	950
Terrestrial feeding	-0.266	-2.025	1.465	950
**Other species-level variables**				
Body mass (scaled)	-0.213	-1.298	0.800	818
Pelagic specialist	0.236	-0.721	1.142	1110
**Random effects**				
Free-flying	33.60	0.002	97.33	950
Ocean basin	0.246	0.003	0.737	950
Source	3.037	2.203	3.926	950
Species	14.86	8.273	20.88	950

B. Excluding pelagic specialist

Pelagic specialists often eat cephalopods (Table S2; correlation = 0.494) so we have repeated the analyses removing the variable "pelagic specialist".

Table S7. Results from a phylogenetic generalised linear mixed model for the frequency of occurrence of plastic (FO) across 230 seabird species with behavioural and life-history traits excluding the pelagic specialist variable. HPD = highest posterior density; ESS = effective sample size. Posterior mode of lambda (λ) = 0.918 (95% HPD: 0.883 - 0.946).

**Fixed effects**	**posterior mean**	**lower 95% HPD**	**upper 95% HPD**	**ESS**
Median year of study	0.564	0.324	0.794	950.000
**Diet**				
Carrion	-0.167	-0.974	0.532	998.570
Cephalopods	0.071	-0.576	0.702	950.000
Crustaceans	0.309	-0.232	0.858	857.001
Fish	-0.061	-1.205	1.015	1068.429
Other invertebrates	0.058	-0.510	0.530	939.413
**Anthropogenic subsidy**				
Fisheries	0.118	-0.413	0.647	853.912
Refuse	-0.324	-1.094	0.492	1045.365
**Foraging method**				
Aerial pursuit	-0.027	-0.810	0.735	950.000
Bottom feeding	-0.822	-2.117	0.502	950.000
Diving	0.306	-0.533	1.182	950.000
Plunging	-0.002	-0.598	0.510	950.000
Scavenging	0.228	-0.438	0.902	861.830
Skimming	0.643	-0.179	1.541	950.000
Surface seizing	-0.013	-0.965	0.979	1302.860
Terrestrial feeding	0.242	-1.141	1.726	950.000
**Other species-level variables**				
Body mass (scaled)	-0.181	-1.125	0.588	712.296
**Random effects**				
Free-flying	7.679	0.002	15.98	950
Ocean basin	0.347	0.006	0.940	950
Source	3.328	2.367	4.237	950
Species	13.28	8.601	18.29	950

C. Excluding terrestrial feeding

Terrestrial feeding is strongly associated with both carrion diets and refuse anthropogenic subsidy (Table S2; correlations = 0.562 and 0.676 respectively) so we have repeated the analyses removing the variable "terrestrial feeding".

Table S8. Results from a phylogenetic generalised linear mixed model for the frequency of occurrence of plastic (FO) across 230 seabird species with behavioural and life-history traits excluding the terrestrial feeding variable. HPD = highest posterior density; ESS = effective sample size. Posterior mode of lambda (λ) = 0.917 (95% HPD: 0.878 - 0.944).

**Fixed effects**	**posterior mean**	**lower 95% HPD**	**upper 95% HPD**	**ESS**
Median year of study	0.564	0.329	0.796	1094
**Diet**				
Carrion	-0.160	-0.915	0.532	950
Cephalopods	0.071	-0.640	0.669	950
Crustaceans	0.287	-0.320	0.797	980
Fish	-0.108	-1.085	0.959	987
Other invertebrates	0.063	-0.391	0.575	950
**Anthropogenic subsidy**				
Fisheries	0.117	-0.429	0.625	861
Refuse	-0.295	-1.181	0.461	1121
**Foraging method**				
Aerial pursuit	0.007	-0.687	0.761	950
Bottom feeding	-0.813	-2.104	0.597	950
Diving	0.362	-0.500	1.213	965
Plunging	0.010	-0.608	0.548	1104
Scavenging	0.291	-0.378	0.932	950
Skimming	0.652	-0.207	1.578	950
Surface seizing	0.019	-0.902	0.936	950
**Other species-level variables**				
Body mass (scaled)	-0.146	-1.023	0.708	827
Pelagic specialist	-0.024	-0.783	0.611	862
**Random effects**				
Free-flying	6.891	0.003	18.40	950
Ocean basin	0.330	0.012	0.957	950
Source	3.315	2.446	4.320	950
Species	13.13	8.583	17.69	950

D. Including nestbound birds

In the full models we excluded nest bound birds as these cannot forage for themselves, and thus their ingested plastics reflect parental foraging rather than individual exposure. It was theorized that our results may therefore under-represent species where most available data come from chicks or fledglings (e.g., sableflesh-footed shearwaters, *Ardenna carneipes*). Here we present the results when nestbound birds are included. Note that this adds only one species. The results are qualitatively identical to those excluding the nest-bound birds, i.e., the main inferences remain the same.

Table S9. Results from a phylogenetic generalised linear mixed model for the frequency of occurrence of plastic (FO) across 231 seabird species with behavioural and life-history traits, including nestbound birds. HPD = highest posterior density; ESS = effective sample size. Posterior mode of lambda (λ) = 0.908 (95% HPD: 0.878 - 0.943).

**Fixed effects**	**posterior mean**	**lower 95% HPD**	**upper 95% HPD**	**ESS**
Median year of study	0.555	0.301	0.786	950
**Diet**				
Carrion	-0.119	-0.817	0.656	848
Cephalopods	-0.006	-0.676	0.668	1061
Crustaceans	0.303	-0.306	0.828	1045
Fish	-0.200	-1.178	0.893	1370
Other invertebrates	0.112	-0.423	0.611	1063
**Anthropogenic subsidy**				
Fisheries	0.205	-0.432	0.714	950.000
Refuse	-0.383	-1.258	0.496	950.000
**Foraging method**				
Aerial pursuit	0.099	-0.654	0.905	950.000
Bottom feeding	-0.750	-2.001	0.726	950.000
Diving	0.480	-0.416	1.384	950.000
Plunging	-0.039	-0.608	0.547	950.000
Scavenging	0.192	-0.545	0.869	950.000
Skimming	0.725	-0.193	1.719	950.000
Surface seizing	0.070	-0.872	1.113	950.000
Terrestrial feeding	0.193	-1.333	1.770	950.000
**Other species-level variables**				
Body mass (scaled)	-0.027	-0.714	0.598	792.910
Pelagic specialist	-0.064	-0.779	0.650	950.000
**Random effects**				
Free-flying	4.439	0.008	6.525	950
Ocean basin	0.322	0.004	0.897	950
Source	3.391	2.543	4.291	950
Species	14.18	9.056	19.37	950

E. Excluding species that were added to the phylogeny (see Table S1)

Table S10. Results from a phylogenetic generalised linear mixed model for the frequency of occurrence of plastic (FO) across 214 species with behavioural and life-history traits using the unmodified BBtree3 rather than the modified version with additional taxa described in Table S1. HPD = highest posterior density; ESS = effective sample size. Posterior mode of lambda (λ) = 0.923 (95% HPD: 0.890 - 0.950).

**Fixed effects**	**posterior mean**	**lower 95% HPD**	**upper 95% HPD**	**ESS**
Median year of study	0.563	0.341	0.788	950
**Diet**				
Carrion	-0.171	-0.822	0.631	973
Cephalopods	0.058	-0.634	0.701	1513
Crustaceans	0.333	-0.285	0.947	950
Fish	-0.030	-1.044	1.061	950
Other invertebrates	-0.017	-0.543	0.480	1027
**Anthropogenic subsidy**				
Fisheries	0.124	-0.423	0.654	950
Refuse	-0.358	-1.238	0.491	950
**Foraging method**				
Aerial pursuit	0.006	-0.796	0.757	950
Bottom feeding	-0.512	-1.914	0.958	950
Diving	0.276	-0.597	1.182	733
Plunging	-0.061	-0.674	0.493	950
Scavenging	0.122	-0.702	0.817	950
Skimming	0.624	-0.241	1.574	1111
Surface seizing	-0.032	-0.934	0.950	950
Terrestrial feeding	0.281	-1.259	1.650	950
**Other species-level variables**				
Body mass (scaled)	-0.191	-1.197	0.725	845
Pelagic specialist	0.010	-0.709	0.804	950
**Random effects**				
Free-flying	9.057	0.002	22.33	950
Ocean basin	0.269	0.007	0.795	839
Source	3.192	2.301	4.135	950
Species	14.34	9.865	20.04	686

F. *Excluding studies where fewer than 10 individuals were sampled*

Table S11. Results from a phylogenetic generalised linear mixed model for the frequency of occurrence of plastic (FO) across 179 species with behavioural and life-history traits using only studies where 10 or more individual birds were sampled. HPD = highest posterior density; ESS = effective sample size. Posterior mode of lambda (λ) = 0.927 (95% HPD: 0.880 - 0.956).

**Fixed effects**	**posterior mean**	**lower 95% HPD**	**upper 95% HPD**	**ESS**
Median year of study	0.624	0.382	0.874	1053
**Diet**				
Carrion	-0.091	-0.897	0.805	1123
Cephalopods	-0.345	-1.082	0.448	950
Crustaceans	0.229	-0.502	0.846	950
Fish	0.707	-0.519	2.148	950
Other invertebrates	0.214	-0.426	0.794	734
**Anthropogenic subsidy**				
Fisheries	0.180	-0.422	0.928	950
Refuse	-0.664	-1.724	0.301	950
**Foraging method**				
Aerial pursuit	-0.041	-0.975	0.947	1046
Bottom feeding	-0.426	-1.903	1.113	950
Diving	0.523	-0.444	1.492	950
Plunging	-0.112	-0.765	0.580	950
Scavenging	0.707	-0.074	1.590	950
Skimming	0.535	-0.460	1.525	1045
Surface seizing	-0.105	-1.330	1.073	950
Terrestrial feeding	-0.227	-1.783	1.353	950
**Other species-level variables**				
Body mass (scaled)	-0.300	-1.461	0.716	806
Pelagic specialist	0.147	-0.655	1.086	1053
**Random effects**				
Free-flying	26.59	0.002	90.39	950
Ocean basin	0.221	0.003	0.730	950
Source	3.108	2.226	4.032	950
Species	13.93	8.023	20.02	950

Order-level results

A. Charadriiformes

Table S12. Results from a phylogenetic generalised linear mixed model for the frequency of occurrence of plastic (FO) across 74 Charadriiformes species with behavioural and life-history traits. HPD = highest posterior density; ESS = effective sample size. Posterior mode of lambda (λ) = 0.951 (95% HPD: 0.889 - 0.982).

**Fixed effects**	**posterior mean**	**lower 95% HPD**	**upper 95% HPD**	**ESS**
Median year of study	0.619	0.131	1.076	935
**Diet**				
Carrion	-2.191	**-4.199**	**-0.113**	827.670
Cephalopods	0.136	-1.140	1.288	950.000
Crustaceans	0.413	-0.874	1.737	1177.014
Fish	1.043	-2.529	4.481	819.483
Other invertebrates	0.400	-1.159	1.948	950.000
**Anthropogenic subsidy**				
Fisheries	0.194	-1.067	1.661	950.000
Refuse	0.159	-0.883	1.448	950.000
**Foraging method**				
Aerial pursuit	0.450	-0.757	1.685	950.000
Bottom feeding	0.531	-2.305	3.508	962.848
Diving	-1.359	-6.261	3.689	1058.625
Plunging	0.759	-0.250	1.828	800.366
Scavenging	0.365	-3.548	3.995	833.926
Skimming	1.315	-2.190	5.146	950.000
Surface seizing	-0.982	-3.232	1.001	950.000
Terrestrial feeding	-0.157	-4.119	4.270	846.553
**Other species-level variables**				
Body mass (scaled)	3.421	-3.931	10.677	950.000
Pelagic specialist	-0.432	-1.332	0.511	950.000
**Random effects**				
Free-flying	1.565	0.002	4.675	950
Ocean basin	0.446	0.003	1.560	950
Source	4.243	2.199	6.237	860
Species	20.97	7.965	39.17	836

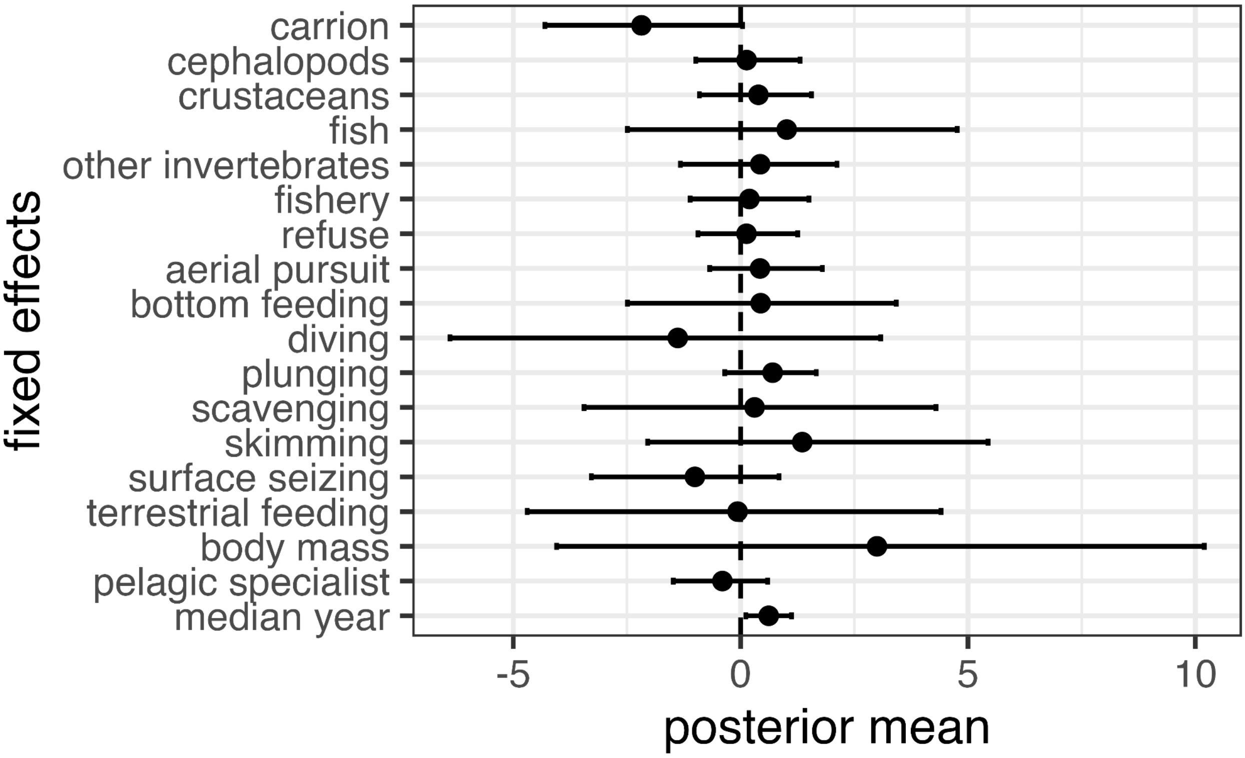

Figure S15. Posterior means and 95% HPD (highest posterior density) intervals for each fixed effect in a phylogenetic generalised mixed model for the frequency of occurrence of plastic (FO) across the 74 Charadriiformes species in our models. The dashed line indicates a posterior mean of 0.

B. Procellariiformes

Table S13. Results from a phylogenetic generalised linear mixed model for the frequency of occurrence of plastic (FO) across 101 Procellariiformes species with behavioural and life-history traits. HPD = highest posterior density; ESS = effective sample size. Posterior mode of lambda (λ) = 0.833 (95% HPD: 0.718 - 0.897). The pelagic specialist variable was excluded because the variable is invariant for this order.

**Fixed effects**	**posterior mean**	**lower 95% HPD**	**upper 95% HPD**	**ESS**
Median year of study	0.441	0.202	0.699	1155
**Diet**				
Carrion	0.354	-0.635	1.370	950
Cephalopods	-0.420	-2.315	1.285	950
Crustaceans	-0.241	-1.095	0.713	950
Fish	-0.671	-1.899	0.563	950
Other invertebrates	0.171	-0.446	0.911	1159
**Anthropogenic subsidy**				
Fisheries	0.160	-0.616	0.864	1087
Refuse	-0.153	-4.420	3.917	950
**Foraging method**				
Aerial pursuit	-2.188	**-4.170**	**-0.400**	850
Bottom feeding	-2.847	-7.529	1.366	732
Diving	0.951	-0.138	1.865	950
Plunging	-0.415	-1.336	0.585	950
Scavenging	0.339	-0.451	1.088	856
Skimming	0.804	-0.252	1.800	950
Surface seizing	0.692	-1.124	2.480	816
Terrestrial feeding	-1.176	-3.841	1.001	950
**Other species-level variables**				
Body mass (scaled)	0.100	-1.809	1.902	845
**Random effects**				
Free-flying	5.395	0.002	13.55	950
Ocean basin	0.283	0.004	0.873	950
Source	2.295	1.524	3.182	950
Species	5.243	2.784	7.869	1324

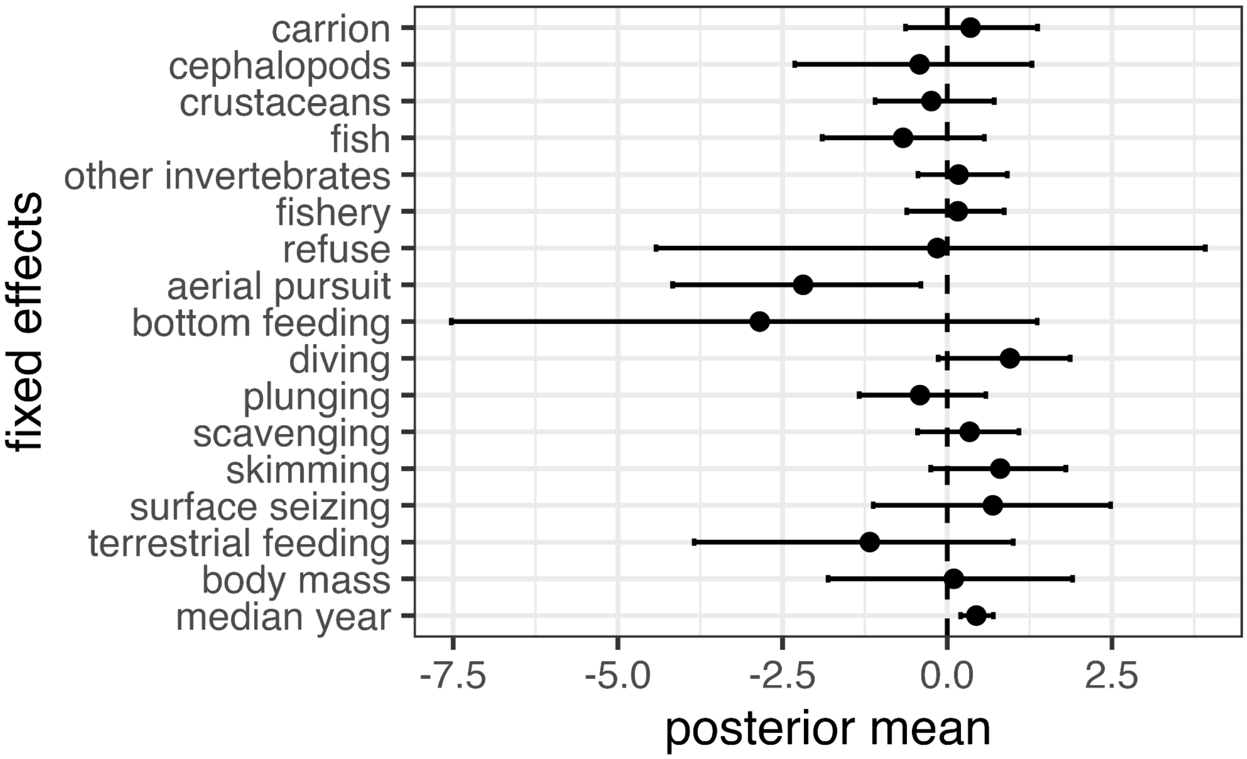

Figure S16. Posterior means and 95% HPD (highest posterior density) intervals for each fixed effect in a phylogenetic generalised mixed model for the frequency of occurrence of plastic (FO) across the 101 Procellariiformes species in our models. The dashed line indicates a posterior mean of 0. The pelagic specialist variable was excluded because the variable is invariant for this order.

Supplementary References

1. Avery-Gomm S *et al.* 2025 Global seabird plastic ingestion database. Natural History Museum. (doi:10.5519/HEMRJX9Q)

2. Claramunt S, Sheard C, Brown JW, Cortés-Ramírez G, Cracraft J, Su MM, Weeks BC, Tobias JA. 2025 A new time tree of birds reveals the interplay between dispersal, geographic range size, and diversification. *Curr. Biol.* **35**, 3883–3895.e4.

3. Clements, J. F., P. C. Rasmussen, T. S. Schulenberg, M. J. Iliff, T. A. Fredericks, J. A. Gerbracht, D. Lepage, A. Spencer, S. M. Billerman, B. L. Sullivan, M. Smith, and C. L. Wood. 2025 The eBird/Clements checklist of Birds of the World: v2025. Downloaded from https://www.birds.cornell.edu/clementschecklist/download/.

4. Gaston AJ. 2004 *Seabirds: a natural history*. Yale University Press, New Haven.

5. Billerman, S. M., B. K. Keeney, G. M. Kirwan, F. Medrano, N. D. Sly, and M. G. Smith, editor. 2025 *Birds of the world*. Cornell Laboratory of Ornithology, Ithaca, NY, USA.

6. Jetz W, Thomas GH, Joy JB, Hartmann K, Mooers AO. 2012 The global diversity of birds in space and time. *Nature* **491**, 444–448.

7. Laist DW. 1997 Impacts of marine debris: Entanglement of marine life in marine debris including a comprehensive list of species with entanglement and ingestion records. In *Marine Debris*, pp. 99–139. New York, NY: Springer New York.

8. Provencher JF, Bond AL, Mallory ML. 2015 Marine birds and plastic debris in Canada: a national synthesis and a way forward. *Environ. Rev.* **23**, 1–13.

9. Ryan PG. 2015 A Brief History of Marine Litter Research. In *Marine Anthropogenic Litter* (ed Bergmann, M., Gutow, L., Klages, M.), pp. 1–25. Cham, Switzerland: Springer International Publishing.

10. Tekman, M.B., Gutow, L., Peter, C., and Bergmann, M. 2021 litterbase.org. Litterbase: Online Portal for Marine Litter. Alfred Wegener Institute Helmholtz Centre for Polar and Marine Research.

11. Tavares DC, de Moura JF, Merico A, Siciliano S. 2017 Incidence of marine debris in seabirds feeding at different water depths. *Mar. Pollut. Bull.* **119**, 68–73.

12. Roman L, Schuyler QA, Hardesty BD, Townsend KA. 2016 Anthropogenic debris ingestion by avifauna in eastern Australia. *PLoS One* **11**, e0158343.

13. Nicastro KR, Lo Savio R, McQuaid CD, Madeira P, Valbusa U, Azevedo F, Casero M, Lourenço C, Zardi GI. 2018 Plastic ingestion in aquatic-associated bird species in southern Portugal. *Mar. Pollut. Bull.* **126**, 413–418.
